# Factors associated with reduction in quality of life after SARS-CoV-2 infection

**DOI:** 10.1038/s41598-025-91388-z

**Published:** 2025-02-26

**Authors:** Christian Neumann, Tim J. Hartung, Klara Boje, Thomas Bahmer, Julian Keil, Wolfgang Lieb, Katrin Franzpoetter, Julius Welzel, Irina Chaplinskaya-Sobol, Matthias Endres, Johanna Geritz, Karl Georg Haeusler, Peter Heuschmann, Andreas Hinz, Sina M. Pütz, Anna Schäfer, Carolin Nuernberger, Lena Schmidbauer, Michael Krawczak, Anne-Kathrin Ruß, Lilian Krist, Thomas Keil, Jennifer Kudelka, Corina Maetzler, Anja Mehnert-Theuerkauf, Felipe A. Montellano, Caroline Morbach, Sein Schmidt, Jan Heyckendorf, Flo Steigerwald, Stefan Stoerk, Christina Lemhoefer, Stefan Schreiber, Carsten Finke, Walter Maetzler

**Affiliations:** 1 Neurology Department, University Medical Center Schleswig-Holstein, Campus Kiel, Arnold-Heller-Str. 3, 24105 Kiel, Germany; 2 Department of Neurology and Experimental Neurology, Charité-Universitätsmedizin Berlin, Bonhoefferweg 3, 10117 Berlin, Germany; 3 Department of Medical Psychology and Medical Sociology, University Medical Center Leipzig, Philipp-Rosenthal-Str. 55, 04103 Leipzig, Germany; 4 Department of General Psychology I and Biological Psychology, Christian-Albrechts-Universität Zu Kiel, Neufeldstraße 4a, 24118 Kiel, Germany; 5 Internal Medicine Department I, Leibniz Lung Clinic, University Hospital Schleswig Holstein, Campus Kiel, Arnold-Heller-Str. 3, 24105 Kiel, Germany; 6 Airway Research Center North (ARCN), German Center for Lung Research (DZL), Woehrendamm 80, 22927 Grosshansdorf, Germany; 7 Institute for Epidemiology, Christian-Albrechts-University Kiel, Niemannsweg 11, 24105 Kiel, Germany; 8 Department of Medical Informatics, University Medical Center Goettingen, Von-Siebold-Str. 3, 37075 Goettingen, Germany; 9 Center for Stroke Research Berlin, Charitéplatz 1, 10117 Berlin, Germany; 10 Excellence Cluster NeuroCure, Charitéplatz 1, 10117 Berlin, Germany; 11 German Center for Neurodegenerative Diseases (DZNE), partner site Berlin, Charitéplatz 1, 10117 Berlin, Germany; 12 German Center for Cardiovascular Research (DZHK), partner site Berlin, Charitéplatz 1, 10117 Berlin, Germany; 13 German Center for Mental Health (DZPG), partner site Berlin, Charitéplatz 1, 10117 Berlin, Germany; 14 Department of Neurology, University of Ulm, Oberer Eselsberg 45, 89081 Ulm, Germany; 15 Institute of Clinical Epidemiology and Biometry, University of Wuerzburg, Josef-Schneider-Str. 2, 97080 Wuerzburg, Germany; 16 Clinical Trial Center, University Hospital Wuerzburg, Oberduerrbacher Str. 6, 97080 Wuerzburg, Germany; 17 Institute for Medical Data Science, University Hospital Wuerzburg, Josef-Schneider-Str. 2, 97080 Wuerzburg, Germany; 18 Department I of Internal Medicine, Center for Integrated Oncology Cologne, University of Cologne, Faculty of Medicine and University Hospital Cologne, Kerpener Str. 62, Cologne, Germany; 19 Institute of Medical Informatics and Statistics, Kiel University, University Medical Center Schleswig-Holstein Campus Kiel, Arnold-Heller-Str. 3, 50937 Cologne, Germany; 20 Institute of Social Medicine, Epidemiology and Health Economics, Charité-Universitätsmedizin Berlin, Schumannstr. 20, 10117 Berlin, Germany; 21 Department Clinical Research and Epidemiology, Comprehensive Heart Failure Center, University Hospital Wuerzburg, Am Schwarzenberg 15, 97080 Wuerzburg, Germany; 22 Department for Medicine I, University Hospital Wuerzburg, Oberduerrbacher Str. 6, 97080 Wuerzburg, Germany; 23 Clinical Study Center, Berlin Institute of Health at Charité – Universitätsmedizin Berlin, Anna-Louisa-Karsch-Straße 2, 10178 Berlin, Germany; 24 Institute of Physical and Rehabilitation Medicine, Jena University Hospital/Friedrich-Schiller-University Jena, Am Klinikum 1, 07747 Jena, Germany

**Keywords:** COVID-19, Post-acute COVID-19 syndrome, Quality of life, Fatigue, EQ-5D-5L, Age

## Abstract

Long-term changes in health-related quality of life (HrQoL) after SARS-CoV-2 infection are common, but their causes and consequences are poorly understood. This prospective, population-based study examined associations between HrQoL and 49 demographic and clinical variables. HrQoL was assessed using the European Quality-of-Life-5-Dimensions-5-Level-Version in 3,475 participants (56% female; aged 18–88 years) approximately 9 months (baseline) and 26 months (follow-up) after their initial infection. Results were compared with the demographic and clinical variables using recursive feature elimination and random forest regression analyses. A statistically significant improvement in HrQoL was observed during the observation period. At baseline, 39% of the variance in HrQoL was explained by fatigue, muscle pain, number of remaining symptoms (RS), perceived stress, and age. At follow-up, fatigue, RS, perceived stress, muscle and joint pain, and age explained 54% of the variance in HrQoL. Changes in HrQoL were associated with changes in fatigue, RS, and perceived stress, meaning that if these decreased from baseline to follow-up, then HrQoL was improved. However, it was not possible to predict whether an individual’s HrQoL would improve or worsen 1 year later based on baseline variable scores. The aforementioned symptoms are specifically associated with impairment in the population’s usual activities.

**Supplementary Information:**

The online version contains supplementary material available at 10.1038/s41598-025-91388-z.

## Introduction

Severe acute respiratory syndrome coronavirus type 2 (SARS-CoV-2) has caused more than 777 million infections and more than 7 million infection-related deaths worldwide as of December 2024^1^. The average recovery time from acute SARS-CoV-2 infection is two to four weeks^2^, and any severe persistence of symptoms for more than 12 weeks is referred to as “post-COVID Syndrome” (PCS)^3,4^ or sometimes as “Long COVID”^5^. The prevalence of PCS in the USA is 14%, and up to 31% of COVID-19 patients developed PCS^6^. Since a large number of people were infected with SARS-CoV-2, these circumstances resulted in a large number of people suffering from PCS, which has since been recognized as a pressing public health issue. Not surprisingly, health-related quality of life (HrQoL) has been found to be reduced in PCS patients^7^. This is also associated with a loss of ability to work, which has economic consequences^8,9^. Although the course of HrQoL in relation to PCS is not well understood, it is likely to be particularly related to fatigue and perceived stress^10,11^. But there is also a wide variety of symptoms that can occur with PCS, including common signs of infection; dermatologic, gastrointestinal, neurologic, and throat disorders; various types of pain; chemosensory deficits; and exercise intolerance^3^.

Fatigue is defined as the depletion of energy reserves combined with an increased need for rest disproportionate to the previous exertion^12^. It appears to be a complex distortion of the physical, mental, cognitive, emotional, motivational, and social states of an individual, resulting in decreased functioning^13,14^. Fatigue has been found to be significantly associated with a decrease in quality of life in many diseases, including multiple sclerosis, cancer, and chronic obstructive pulmonary disease^15–17^. Fatigue is also one of the most commonly reported symptoms of PCS^18^, affecting 71% of patients^19^. Fatigue in the context of PCS has been associated with distinct structural and functional brain changes^20–22^. Depression and anxiety were also shown to be risk factors for prolonged fatigue after SARS-CoV-2 infection^23^. The relationship between HrQoL and perceived stress has been shown in other lung diseases, such as asthma^24^. In the case of SARS-CoV-2, people who had increased perceived stress before infection were more likely to report PCS symptoms that interfered with daily life after infection^25^. Thus, higher perceived stress may be a risk factor for the maintenance of PCS symptoms. In addition, PCS patients with symptoms such as fatigue, weakness, dyspnea, shortness of breath, palpitations, forgetfulness, numbness, sleep disturbance, cough, chest pain, muscle pain, joint pain, headache, anosmia, hair loss, taste loss, and many others have significantly lower HrQoL^26–28^. However, studies disagree on which symptoms actually significantly worsen HrQoL, and it is also largely unclear how much a given symptom worsens HrQoL and in what constellation it does so.

In summary, psychological and psychosocial factors, as well as physical symptoms, play a role in the well-being of former SARS-CoV-2 patients. There are an overwhelming number of symptoms that can occur in PCS, in different combinations and with different degrees of severity. However, to the best of our knowledge, there is no literature on which symptoms in combination are affecting HrQoL the most. The question is which symptoms are most detrimental to HrQoL and whether there are any symptoms that have no detrimental effect on HrQoL at all. This information would be essential to prioritize certain aspects of patients in the most efficient way for multimodal therapy approaches. This study aims to examine a large number of factors and symptoms in an exploratory way to determine which are most strongly associated with reduced HrQoL and the intensity with which they affect the HrQoL. To our knowledge, this is the first large-scale prospective study of the effect of demographic and clinical variables on HrQoL in individuals with and without PCS.

## Methods

### Study cohort

COVIDOM is a prospective, longitudinal, multicenter, population-based study of the long-term sequelae of COVID-19, carried out in Kiel, Berlin, and Wuerzburg under the umbrella of the German National Pandemic Cohort Network (NAPKON). Details about COVIDOM (aka NAPKON-POP) have been published before^25,29,30^, including study protocol, recruitment scheme, cohort characteristics, and non-responder analyses. Briefly, COVIDOM includes individuals aged 18 years or older, resident in Schleswig-Holstein (Kiel region), Berlin-Neukoelln, or Lower Franconia, Germany, and diagnosed SARS-CoV-2 positive by way of a PCR test. Public health authorities supported recruitment into COVIDOM by mailing appropriate invitations to potential participants. The main exclusion criterion was an acute re-infection at the scheduled study site visit. The first assessment (baseline) took place at least 6 months after infection, followed by a second assessment (follow-up) at least 18 months after infection. The present study included COVIDOM participants who underwent a baseline assessment between November 16, 2020, and May 17, 2023, and whose follow-up assessment took place between May 10, 2022, and September 28, 2023. All of the participants were invited to follow-up, but at the time of the data analysis, this had not yet taken place for all of them. In accordance with the Declaration of Helsinki, written informed consent was obtained from all COVIDOM participants. The study has received ethical approval from the respective Ethics Committees (for further information, see the patient consent and ethics approval section of this paper). COVIDOM was registered in the German Registry of Clinical Studies (DRKS00023742) and in ClinicalTrials.gov (NCT04679584).

### Questionnaires & assessments

HrQoL was assessed using the European Quality-of-Life-5-Dimensions-5-Level-Version (EQ-5D-5L)^31^. With this tool, HrQoL is rated along the five dimensions of mobility, self-care, usual activities, pain/discomfort, and anxiety/depression. Each dimension provides five response options: no problems, slight problems, moderate problems, major problems, and extreme problems. Each response contributes to a quality-of-life index between 0 and 1, with 1 representing the best condition and 0 representing the worst condition.

The Functional Assessment of Chronic Fatigue Illness – Fatigue Subscore (FACIT-Fatigue Scale) questionnaire was used to assess fatigue^32^. The FACIT-Fatigue Scale comprises 13 items measuring fatigue symptoms over the previous seven days. A 5-point Likert scale is used to rate the severity of fatigue symptoms as “not at all”, “a little”, “moderate”, “quite”, or “very”. The overall score ranges from 0 to 52, with lower scores indicating more severe symptoms and scores ≤ 30 considered clinically relevant fatigue^33^.

The 10-item Perceived Stress Scale (PSS-10) was used to measure perceived stress, defined as the degree to which life was experienced as unpredictable, uncontrollable and overwhelming^34^. A 5-point Likert scale is used to rate the frequency with which an item was experienced in the past month as “never”, “almost never”, “sometimes”, “fairly often”, or “very often”. The total score ranges from 0 to 40, with higher scores indicating higher levels of perceived stress.

Sociodemographic information was collected using standardized questionnaires. Height and weight were measured at the study site visit. In a general standardized clinical interview, patients were also asked what symptoms they had during their SARS-CoV-2 infection, which of these symptoms persisted to the time of the assessment, what pre-diagnosed diseases they had before their SARS-CoV-2 infection, their vaccination status, their employment status, and whether they were treated during their SARS-CoV-2 infection (at home, in hospital, in intensive care, and/or on mechanical ventilation). In an additional neurology-specific standardized clinical interview, participants were asked whether they subjectively felt cognitively impaired since the infection (i.e., word-finding disorder).

The Montreal Cognitive Assessment (MoCA) was administered to assess global cognition. According to the manual, a score < 26 is considered an indication of cognitive impairment^35^. The MoCA includes information on whether they have > 12 years of education.

### Statistical analysis

R software version 4.3.2^36^ was used for data visualization and statistical analysis. Participants with a missing value or answer “don’t know” for at least one of a pre-specified set of (relevant) variables were excluded from the respective analysis. Details about all variables included in the statistical analysis are provided in Supplementary Table S1.

### Recursive feature elimination (RFE)

Regression modeling of the HrQoL of participants was performed separately for the baseline and follow-up data. Prior to each regression, Recursive Feature Elimination (RFE) served to determine the most efficient number of independent variables to be included in the final modeling. To this end, interim models were computed using a 10-fold cross-validation method implemented in R package caret^37^. RFE starts with a full model and iteratively reduces the number of independent variables by one. The advantage of this method is that it takes into account the interaction of variables in any possible constellation. Models were evaluated exploratory by their associated root-mean-square error (RMSE) to determine, by visual inspection of the RMSE plot, the best compromise between model fit and the number of independent variables included. The selected variables were then included in the final random forest regression analysis. Because we don’t know in advance which variables will be selected by RFE, the following analyses are exploratory, as we made no assumptions about possible effects.

### Random forest regression

Random forest regression analysis was performed to quantify the association between HrQoL, measured by EQ-5D-5L, and the RFE-selected independent variables. Random forest regression is suitable for large datasets with complex interactions and is robust to noisy data and outliers^38,39^. The number of independent variables randomly sampled as candidates at each split was set to two, and the number of trees to be grown was set to 2000. Data were divided randomly into a training set, comprising 70% of the data, and a test set, comprising 30%. The random forest regression models were trained with R package randomForest^40,^ and each resulting model was validated on the test set. The coefficient of determination (*R*^*2*^) and RMSE of the final model were used to estimate the amount of variance of EQ-5D-5L explained and the goodness of fit of each model. The mean decrease in accuracy (MDA) of the variables in the final model was used to quantify the overall importance of each independent variable for the model. Accumulated local effects (ALE) plots were used to illustrate the value-specific effects of each independent variable on EQ-5D-5L. 

### Additional analyses

To ensure that the amount of variance explained and the relevance of the selected variables were not due to psychiatric conditions present in the cohort prior to the pandemic, the procedure described above was repeated in subsets of the data that included only participants with or without a pre-diagnosis of anxiety or depression. The final random forest regression analysis was also repeated with each dimension of the EQ-5D-5L, respectively. In addition, RFE and random forest regression analyses were performed to determine whether a particular combination of independent variables at baseline could predict HrQoL at follow-up. Selected relevant variables were further analyzed by defining the change in each variable as a new independent variable and treating the difference in EQ-5D-5L between baseline and follow-up as the outcome. Spearman rank correlation coefficients served to quantify the relationships between variables. A two-tailed Wilcoxon signed-rank test for paired samples was used to assess whether EQ-5D-5L changed significantly over time. The resulting effect sizes were interpreted following Cohen^41^. *P*-values < 0.05 were considered statistically significant.

## Results

### Study sample

There were 3,475 participants at baseline and 2,510 at follow-up. The median age of the participants was 44 years (quartiles: 32; 57). The median time since initial infection was 299 days (quartiles: 165; 361) at baseline and 744 days (quartiles: 657; 835) at follow-up. Eleven percent of participants reported a previous diagnosis of depression, and 3% suffered from an anxiety disorder prior to their SARS-CoV-2 infection. Twenty percent had clinically relevant fatigue at baseline and 18% at follow-up. Additional sociodemographic and clinical details of the study sample, separated by whether clinically relevant fatigue was found according to FACIT-F, are shown in Table 1, and extended cohort characteristics are shown in Supplementary Table S2.

**Table 1:** Sociodemographic and clinical characteristics of the COVIDOM study sample at baseline for people with clinically relevant fatigue (CRF, FACIT-Fatigue ≤ 30) and without. 266 participants had no FACIT-Fatigue score.

Characteristics	With CRF = 689	Without CRF = 2,520
**Sex**		Missing = 1
Female [N (%)]	461 (67)	1,328 (53)
Male [N (%)]	228 (33)	1,191 (47)
**Age [years]**		
18–34 [N (%)]	190 (28)	825 (33)
35–49 [N (%)]	235 (34)	660 (26)
50–64 [N (%)]	232 (34)	755 (30)
65–88 [N (%)]	32 (5)	280 (11)
**Unemployment**		
Yes [N (%)]	114 (17)	447 (18)
**BMI**	Missing = 11	Missing = 25
Underweight [N (%)]	11 (2)	28 (1)
Normal weight [N (%)]	254 (37)	1064 (42)
Overweight [N (%)]	204 (30)	868 (34)
Obese [N (%)]	209 (30)	535 (21)
**Time between initial SARS-CoV-2 infection and baseline**		
6–9 months [N (%)]	365 (53)	1122 (45)
9–12 months [N (%)]	236 (34)	1005 (40)
≥ 12 months [N (%)]	88 (13)	393 (16)
**Number of remaining symptoms**		
Asymptomatic [N (%)]	187 (27)	1,594 (63)
1–5 [N (%)]	382 (55)	863 (34)
6–8 [N (%)]	85 (12)	49 (2)
9–11 [N (%)]	28 (4)	10 (< 1)
12–21 [N (%)]	7 (1)	4 (< 1)
**Pre-diagnosed COVID-19 comorbidity**		
Any neurological/psychiatric disorder	284 (41)	473 (19)
Depression	170 (25)	179 (7)
Migraine	97 (14)	191 (8)
Anxiety	35 (5)	64 (3)
Apnea	50 (7)	76 (3)
COPD	15 (2)	26 (1)
Tumor	11 (2)	31 (1)

*Note.* BMI, Body-Mass-Index; COPD, chronic obstructive pulmonary disease; EQ-5D-5L, European Quality of Life 5 Dimensions 5 Level Version; FACIT-Fatigue Scale, Functional Assessment of Chronic Fatigue Illness – Fatigue Subscore.

After excluding participants with missing data on any of the 50 variables, baseline data were available for the RFE for 2,174 of the 3,475 participants. Of these, 1,579 also had follow-up data, leaving 1,519 participants after exclusion of cases with missing data (Supplementary Fig. S3), which were used for the prediction analysis. For the RFE at follow-up, data were available for 2,350 of the 2,510 participants after excluding participants with missing data on any of the 29 variables.

### Variables associated with HrQoL

The RMSE plots of all models that were computed in the RFE steps of the analysis (S4 – S9), and the MDA values of the variables that were included in each final model (S10 – S15) are provided in the Supplementary Information. Details of the final random forest regression models are provided in Supplementary Table S16.

### Baseline

RFE identified the following five variables as relevant for inclusion in the final regression analysis of EQ-5D-5L (in descending order, starting with the most relevant variable): Fatigue (FACIT-Fatigue Scale), muscle pain (yes/no), Number of symptoms remaining from initial SARS-CoV-2 infection (RS), perceived stress (PSS), and Age (years). The final regression model could be calculated with 3,117 participants, and it achieved an *R*^*2*^ value of 0.39 (RMSE = 0.11), meaning that 39% of the variance in the EQ-5D-5L could be explained by the model through the five independent variables.

**Fig. 1:**
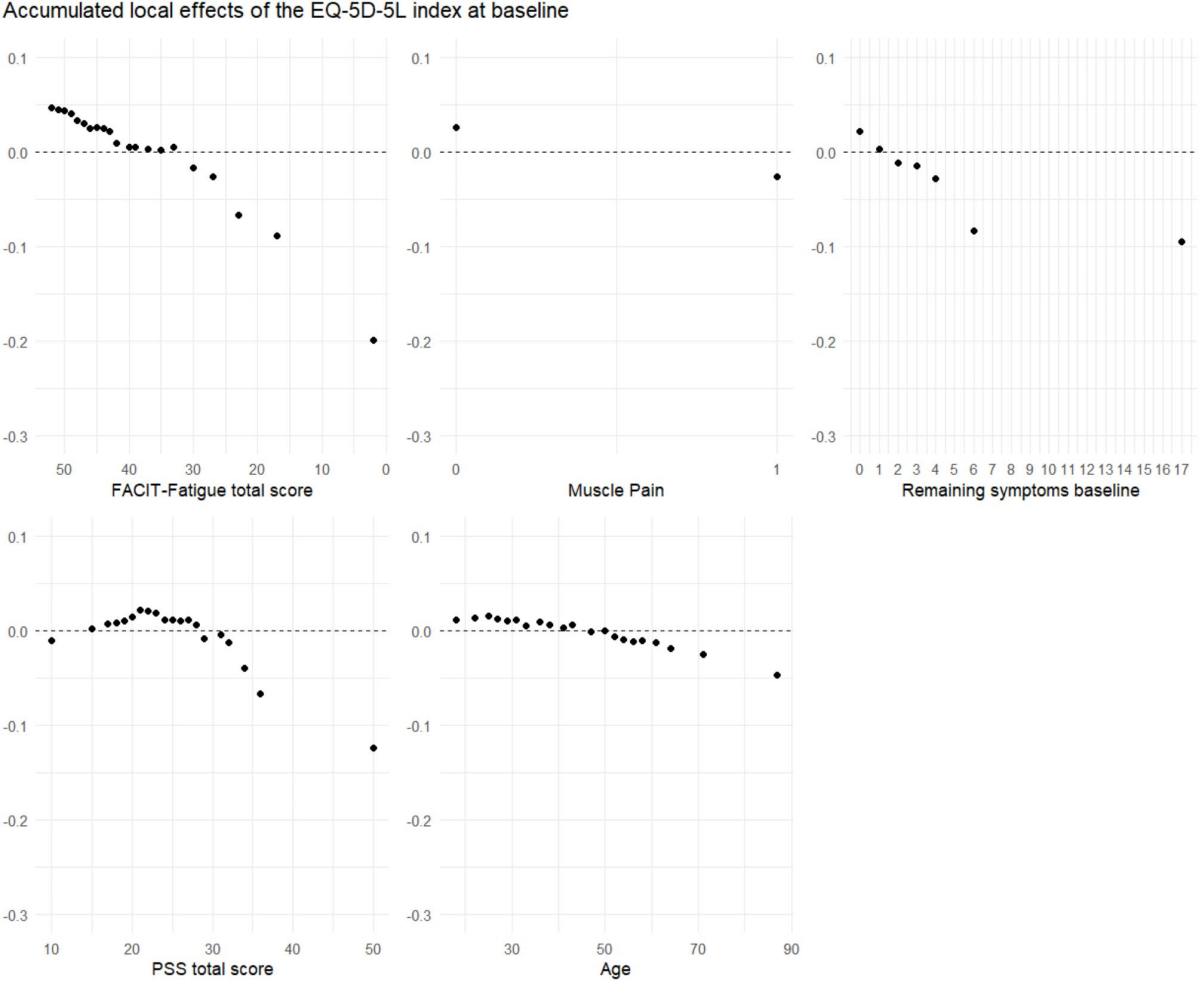
Accumulated local effects (ALE) plots of the independent variables included in the random forest regression analysis of EQ-5D-5L at baseline. The x-axis is the scale of the respective variable, whereas the y-axis measures the average difference between the value-specific and the overall impact of the respective independent variable upon the outcome variable (EQ-5D-5L). Each dot corresponds to a value of the respective independent variable that was observed at least once in the study sample. EQ-5D-5L, European Quality-of-Life-5-Dimensions-5-Level-Version; FACIT-Fatigue Scale, Functional Assessment of Chronic Fatigue Illness – Fatigue Subscore; PSS, Perceived Stress Scale.

In the ALE plots of the variables selected for the analysis (Fig. 1), FACIT-Fatigue Scale ≤ 30, presence of muscle pain, RS > 1, PSS > 28, and age > 50 years were all independently associated with a lower than average EQ-5D-5L index.

In participants without pre-diagnosed depression or anxiety, the same five independent variables were selected by RFE, and the final model accounted for 35% of the variance in EQ-5D-5L (RMSE = 0.09). In terms of their relative importance, as measured by MDA, muscle pain moved from the second to the fifth place. In participants with pre-diagnosed depression or anxiety, fatigue, RS and PSS were sufficient to explain 30% of the variance in EQ-5D-5L (RMSE = 0.15).

Regression models in Supplementary Table S17 using the individual dimension of the EQ-5D-5L instead of the index showed that the selected variables had a similar but slightly lower *R*^*2*^ value in the mobility (*R*^*2*^ = 29%) and anxiety/depression (*R*^*2*^ = 29%) dimensions. With the selected variables, most variance could be explained in the usual activities dimension (*R*^*2*^ = 47%), while the self-care dimension had a small *R*^*2*^ value (*R*^*2*^ = 4%).

### Follow-up

**Fig. 2:**
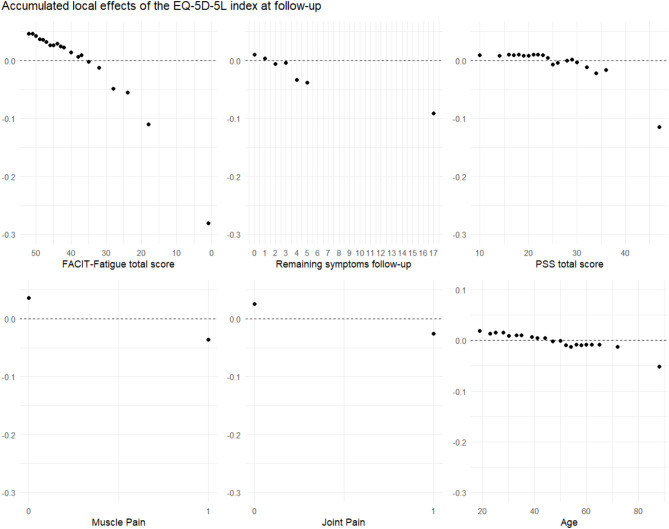
Accumulated local effects (ALE) plots of the independent variables included in the random forest regression analysis of EQ-5D-5L at follow-up. For details, see legend to Fig. 1

RFE identified the following six variables as most important in explaining EQ-5D-5L at follow-up: Fatigue, RS, PSS, muscle pain, oint pain, and Age. The final regression model could be calculated with 2,350 participants, and it explained 54% of the variance of EQ-5D-5L (RMSE = 0.10). The ALE plots (Fig. 2) revealed that FACIT-Fatigue Scale < 35, RS > 3, PSS > 30, the presence of muscle pain or joint pain, and age > 50 were all independently associated with lower than average EQ-5D-5L.

Among participants without pre-diagnosed depression or anxiety, the corresponding model explained 43% of the variance in EQ-5D-5L (RMSE = 0.09), compared to 53% for participants with pre-diagnosed depression or anxiety (RMSE = 0.15). In the final models of both subsets, the same six variables were selected as in the full data set. Age had more weight after the data were separated. In the subgroup with pre-diagnosed depression or anxiety, RS (rank 5) had less weight.

Regression models in Supplementary Table S17 using the individual dimensions of the EQ-5D-5L instead of the index showed that the selected variables had a low *R*^*2*^ value in the mobility (*R*^*2*^ = 23%) and self-care (*R*^*2*^ = 14%) dimensions. The pain/discomfort (*R*^*2*^ = 40%) and anxiety/depression (*R*^*2*^ = 43%) dimensions had a lower *R*^*2*^ value than the index but were still moderately high.

### Changes of HrQoL and independent variables

**Fig. 3:**
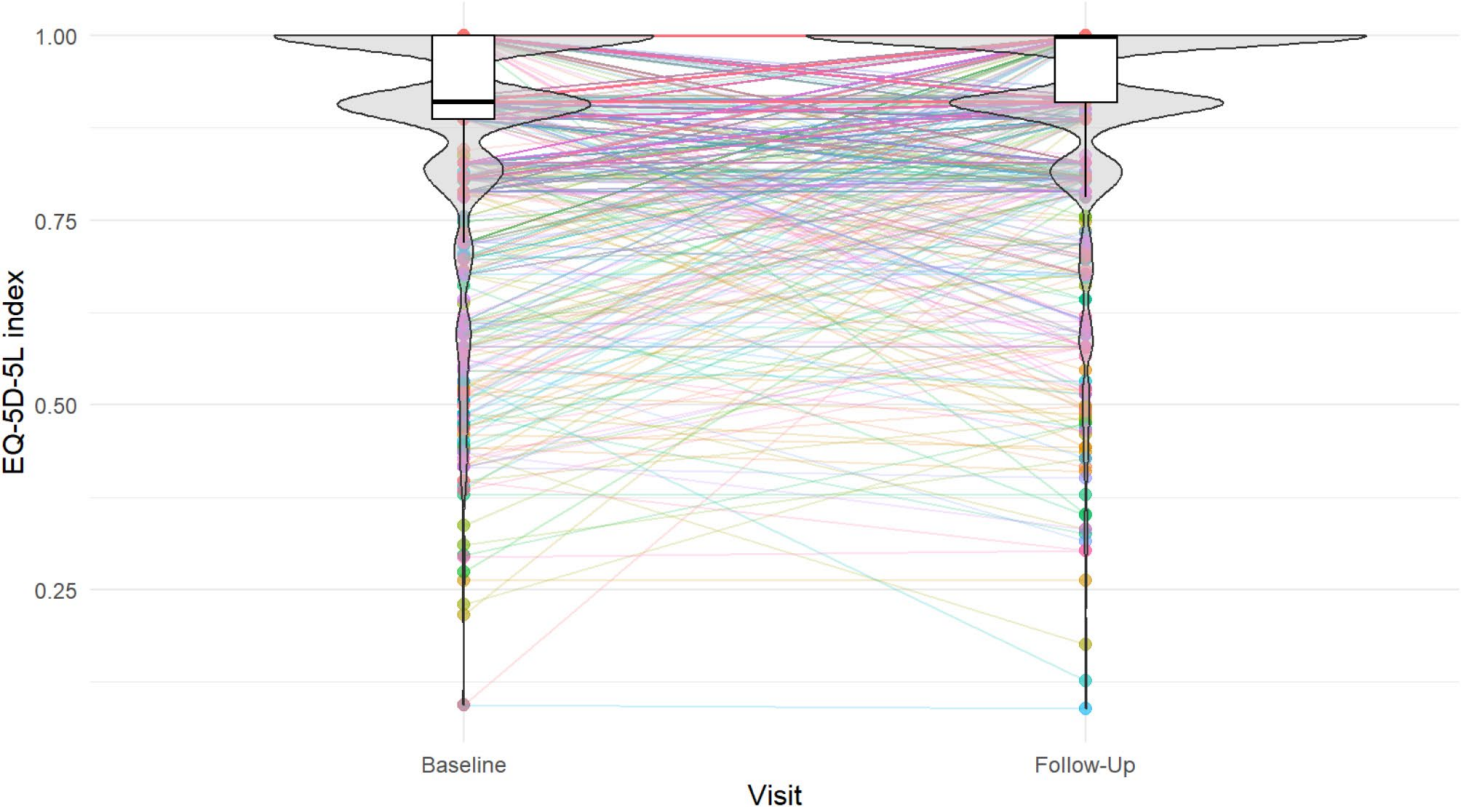
Box plots showing the longitudinal change in the EQ-5D-5L index by displaying median, quartiles, range, and data points for baseline and follow-up, with additional lines to visualize each participant’s trajectory, and violin plots to visualize the density of the respective data. EQ-5D-5L, European Quality-of-Life-5-Dimensions-5-Level-Version.

A difference of 0.09 was observed between the median EQ-5D-5L at baseline (median = 0.91; quartiles = 0.89, 1.00) and follow-up (0.999; 0.91, 1.00; Fig. 3). The two-tailed Wilcoxon signed-rank test showed that this difference was significant, with a small effect size (*r* = 0.23). This indicates that HrQoL increased 1 year after the first assessment in this cohort. However, even when all 49 possible independent variables measured at baseline were considered, the RFE did not result in a regression model that explained more than 1% of the variance of the EQ-5D-5L at follow-up (RMSE = 0.13).

On the other hand, changes from baseline to follow-up in FACIT-Fatigue Scale (Spearman correlation coefficient *r* = 0.27, *p* < 0.001), RS (*r* = -0.23, *p* < 0.001), and PSS (*r* = -0.13, *p* < 0.001) were all significantly correlated with the corresponding change in EQ-5D-5L. A regression model including these three changes explained 11% of the variance in the change in EQ-5D-5L (RMSE = 0.12). Although it was not possible to predict which participants would experience a decrease in HrQoL, it is associated with a decrease in fatigue, remaining symptoms and perceived stress.

## Discussion

This explorative large prospective observational study, covering a 2-year period after SARS-CoV-2 infection, found that the HrQoL of patients is notably affected initially, but improves over time. Fatigue, the number of symptoms remaining from the acute phase of COVID-19, perceived stress, muscle pain, and age were found to play a role in HrQoL at both the beginning and at the end of the observation period.

At approximately nine months after acute SARS-CoV-2 infection, 39% of the variance in HrQoL could be explained in the present study by just five variables. This is a remarkably strong relationship between HrQoL and fatigue, muscle pain, the number of symptoms remaining from the acute phase of COVID-19, perceived stress, and age. Notably, fatigue had the strongest association with HrQoL (both at baseline and follow-up). Specifically, participants with a FACIT-Fatigue ≤ 30 have below-average HrQoL, further supporting the cut-off used to diagnose clinically relevant fatigue. These results fit well with other studies that have identified fatigue as the most important variable for PCS. For example, a score has been developed to assess the severity of PCS, with fatigue being a symptom with the highest weight in the calculation of this score^3^. This PCS score also correlates with HrQoL. Although most studies agree that fatigue is the most common symptom in PCS^26–28^, none of them report the extent to which it affects HrQoL. The fact that fatigue appears to be the most common symptom and also the one that most affects HrQoL emphasizes the fact that the treatment of PCS consists primarily of the treatment of fatigue. But it is more complex than that, because treatment options that would be classically chosen for fatigue, such as physical activity^42^, may be contraindicated for people with muscle pain. Another study^27^ supports the notion that muscle pain is one of the strongest associations with HrQoL, as in this case.

Patients with at least two persistent symptoms appeared to suffer from reduced HrQoL at baseline. This suggests that even 9 months after the acute infection, the persistence of symptoms that occurred at that time had an influence on HrQoL. There is also likely to be an inverse effect: The lower the HrQoL prior to infection, the greater the likelihood of experiencing an increased number of symptoms that persist after infection. One indication of this is that people with no residual symptoms from the initial infection have an above-average HrQoL in this sample, even though they make up 55% of the sample (supplementary table S2). From a causal perspective, it is unlikely that the absence of residual symptoms would raise the HrQoL of individuals above the average; therefore it is possible that people with a high HrQoL are less likely to have symptoms up to 2 years after SARS-CoV-2 infection. This may indicate that healthy adults were less affected by the virus than those who already had some HrQoL-reducing conditions, as suggested in the literature^43^. The importance of perceived stress also makes sense because it may be a risk factor for an increased number of symptoms^25^, and having new symptoms may increase your perceived stress. Finally, it is also commonly reported that people of older age are more negatively affected by the infection^44,45^. We would suggest that people > 50 have a higher risk for decreased HrQoL.

To examine whether pre-existing depressive or anxiety disorder acted as a confounder of the above association with HrQoL, the analysis was stratified according to these conditions. The effect of the identified variables on HrQoL was found to be largely independent of whether or not patients had either disorder at the time of acute SARS-CoV-2 infection or not. All regression models explained at least a moderate amount of variance in HrQoL, and fatigue remained the most important variable. For participants with pre-diagnosed depression or anxiety, the importance of fatigue appears to be greater than the other variables by a greater margin than for those without (Supplementary Fig. S14). The co-occurrence of fatigue and depression is a known phenomenon and might explain this result^46^. This highlights these comorbidities as potential risk factors for fatigue progression, as suggested by other studies^25^.

Using the individual dimensions of the EQ-5D-5L, it was found that the selected variables were specifically associated with the Usual Activities dimension. Since chronic fatigue makes usual activities of daily living more difficult, it makes sense that fatigue (probably caused by the infection) would be the cause of the decline in HrQoL.

At 26 months, on average, fatigue was found to be a covariate of HrQoL with an even stronger association, i.e., fatigue becomes increasingly important in reducing HrQoL over time. This is probably the main reason why the amount of explained variance in HrQoL increased to 54% at follow-up, using only one more variable (joint pain). This again highlights the long-term role of fatigue in people after a SARS-CoV-2 infection, as HrQoL is mostly dependent on the intensity of this symptom. At follow-up, the effect of the remaining symptoms seems to be similar, but the decrease in HrQoL for participants with 2 or 3 symptoms became smaller. This could be explained by habituation to the few symptoms they have. The importance of muscle pain appears to be less than at baseline, which would be a positive message for patients that this symptom is less important to HrQoL over time.

The association of the selected variables with the anxiety/depression dimension increased at follow-up, suggesting that mental health becomes more affected over time. This makes sense because it is expected that worry, hopelessness, and social problems will increase the longer the illness lasts. Interestingly, the number of symptoms remaining from the acute phase of COVID-19 became remarkably less important in participants with pre-diagnosed depression or anxiety at follow-up. This further emphasizes that the course of PCS is different in patients with pre-diagnosed depression or anxiety. Treatment should be tailored to the individual pattern of illness. For example, although the majority of PCS patients would likely benefit from psychotherapy, patients with a pre-diagnosed psychiatric illness need a different approach than those without.

In the present study, HrQoL showed a significant improvement from baseline to follow-up. This change was also accompanied by improvements in fatigue, number of symptoms remaining from the acute phase of COVID-19, and perceived stress. However, the best-fitting regression model could not predict more than 1% of the variance in HrQoL improvement from the baseline values of its covariates. This means that the HrQoL at different stages of the recovery from COVID-19 is more dependent on a patient’s current health status than on their status at the onset of PCS. It can be concluded that, although predicting the degree of improvement in HrQoL is complex and requires further research, patients who experience fatigue, a high number of symptoms, stress, and muscle pain at 9 months after SARS-COV-2 infection can still expect their HrQoL to improve if these symptoms improve in the following months.

The study has the following limitations: Many participants had to be excluded due to missing data. Any participant with even one missing value was excluded to ensure a more robust analysis. Although the number of participants is still large enough, it cannot be completely excluded that participants with a higher number of symptoms have missing values, which could lead to bias. Furthermore, it is possible that the magnitude of the association is overestimated because it cannot be proven that all reported symptoms are directly attributable to SARS-CoV-2 infection. However, there is no doubt that they occurred in temporal association with the infection.

In conclusion, the present study showed that HrQoL improved significantly during the first 2 years after SARS-CoV-2 infection and that fatigue, perceived stress and number of remaining symptoms were the main determinants of poor HrQoL, followed by muscle pain, age and joint pain. To improve the HrQoL of PCS patients, future clinical research in this area should therefore focus on the prevention of chronic fatigue, which is the most debilitating and common symptom of PCS and currently has the fewest treatment options. In addition, depression and anxiety as comorbidities should also be taken seriously in the management of PCS patients, as they are important for the HrQoL of patients even 26 months after the initial SARS-CoV-2 infection.

## Electronic supplementary material

Below is the link to the electronic supplementary material.

Supplementary Material 1

## Data Availability

The datasets used and/or analysed during the current study available from uac@nukleus.netzwerk-universitaetsmedizin.de on reasonable request. More information is available on the NAPKON website (https://proskive.napkon.de/).
