# Correction: Factors associated with reduction in quality of life after SARS-CoV-2 infection

**DOI:** 10.1038/s41598-026-58304-5

**Published:** 2026-06-17

**Authors:** Christian Neumann, Tim J. Hartung, Klara Boje, Thomas Bahmer, Julian Keil, Wolfgang Lieb, Katrin Franzpoetter, Julius Welzel, Irina Chaplinskaya-Sobol, Matthias Endres, Johanna Geritz, Karl Georg Haeusler, Peter Heuschmann, Andreas Hinz, Sina M. Pütz, Anna Schäfer, Carolin Nuernberger, Lena Schmidbauer, Michael Krawczak, Anne-Kathrin Ruß, Lilian Krist, Thomas Keil, Jennifer Kudelka, Corina Maetzler, Anja Mehnert-Theuerkauf, Felipe A. Montellano, Caroline Morbach, Sein Schmidt, Jan Heyckendorf, Flo Steigerwald, Stefan Stoerk, Christina Lemhoefer, Stefan Schreiber, Carsten Finke, Walter Maetzler

**Affiliations:** 1 Neurology Department, University Medical Center Schleswig-Holstein, Campus Kiel, Arnold-Heller-Str. 3, 24105 Kiel, Germany; 2 Department of Neurology and Experimental Neurology, Charité-Universitätsmedizin Berlin, Bonhoefferweg 3, 10117 Berlin, Germany; 3 Department of Medical Psychology and Medical Sociology, University Medical Center Leipzig, Philipp-Rosenthal-Str. 55, 04103 Leipzig, Germany; 4 Department of General Psychology I and Biological Psychology, Christian-Albrechts-Universität Zu Kiel, Neufeldstraße 4a, 24118 Kiel, Germany; 5 Internal Medicine Department I, Leibniz Lung Clinic, University Hospital Schleswig Holstein, Campus Kiel, Arnold-Heller-Str. 3, 24105 Kiel, Germany; 6 Airway Research Center North (ARCN), German Center for Lung Research (DZL), Woehrendamm 80, 22927 Grosshansdorf, Germany; 7 Institute for Epidemiology, Christian-Albrechts-University Kiel, Niemannsweg 11, 24105 Kiel, Germany; 8 Department of Medical Informatics, University Medical Center Goettingen, Von-Siebold-Str. 3, 37075 Goettingen, Germany; 9 Center for Stroke Research Berlin, Charitéplatz 1, 10117 Berlin, Germany; 10 Excellence Cluster NeuroCure, Charitéplatz 1, 10117 Berlin, Germany; 11 German Center for Neurodegenerative Diseases (DZNE), partner site Berlin, Charitéplatz 1, 10117 Berlin, Germany; 12 German Center for Cardiovascular Research (DZHK), partner site Berlin, Charitéplatz 1, 10117 Berlin, Germany; 13 German Center for Mental Health (DZPG), partner site Berlin, Charitéplatz 1, 10117 Berlin, Germany; 14 Department of Neurology, University of Ulm, Oberer Eselsberg 45, 89081 Ulm, Germany; 15 Institute of Clinical Epidemiology and Biometry, University of Wuerzburg, Josef-Schneider-Str. 2, 97080 Wuerzburg, Germany; 16 Clinical Trial Center, University Hospital Wuerzburg, Oberduerrbacher Str. 6, 97080 Wuerzburg, Germany; 17 Institute for Medical Data Science, University Hospital Wuerzburg, Josef-Schneider-Str. 2, 97080 Wuerzburg, Germany; 18 Department I of Internal Medicine, Center for Integrated Oncology Cologne, University of Cologne, Faculty of Medicine and University Hospital Cologne, Kerpener Str. 62, Cologne, Germany; 19 Institute of Medical Informatics and Statistics, Kiel University, University Medical Center Schleswig-Holstein Campus Kiel, Arnold-Heller-Str. 3, 50937 Cologne, Germany; 20 Institute of Social Medicine, Epidemiology and Health Economics, Charité-Universitätsmedizin Berlin, Schumannstr. 20, 10117 Berlin, Germany; 21 Department Clinical Research and Epidemiology, Comprehensive Heart Failure Center, University Hospital Wuerzburg, Am Schwarzenberg 15, 97080 Wuerzburg, Germany; 22 Department for Medicine I, University Hospital Wuerzburg, Oberduerrbacher Str. 6, 97080 Wuerzburg, Germany; 23 Clinical Study Center, Berlin Institute of Health at Charité – Universitätsmedizin Berlin, Anna-Louisa-Karsch-Straße 2, 10178 Berlin, Germany; 24 Institute of Physical and Rehabilitation Medicine, Jena University Hospital/Friedrich-Schiller-University Jena, Am Klinikum 1, 07747 Jena, Germany

Correction to: *Scientific Reports* 10.1038/s41598-025-91388-z, published online 26 February 2025

The original version of this Article contained an error in the spelling of the author Anna Schäfer which was incorrectly given as Anna Horn.

The original Article has been corrected.

