# Supplementary material for: Factors associated with reduction in quality of life after SARS-CoV-2 infection
Source: Sci Rep. 2025 Feb 26;15:6833. doi: 10.1038/s41598-025-91388-z (PMC11861590; doi:10.1038/s41598-025-91388-z)
Supplement: Supplementary file 1 — Supplementary Material 1 [file 41598_2025_91388_MOESM1_ESM.pdf]

Supplementary Information

Factors Associated with Reduction in Quality of Life After SARS-CoV-2 Infection

Christian Neumann, MSc[a*], Tim J. Hartung, MD MA[b,c*], Klara Boje, BSc[d], Thomas Bahmer, MD[e,f], Julian Keil, PhD[d], Wolfgang Lieb, MD MSc[g], Katrin Franzpoetter, MSc[g], Julius Welzel, MSc[a], Irina Chaplinskaya-Sobol, MSc[h], Matthias Endres, MD[b,i,j,k,l,m], Johanna Geritz, PhD[a], Karl Georg Haeusler, MD[n], Peter Heuschmann, MD[o,p,q], Andreas Hinz, PhD[c], Sina Hopff, MD[r], Anna Schäfer[o,q], Carolin Nuernberger, MSc[o,q], Lena Schmidbauer, MSc[o,q], Michael Krawczak, PhD[s], Anne-Kathrin Ruß, MSc[g,s], Lilian Krist, MD[s], Thomas Keil[t], Jennifer Kudelka, MD[a], Corina Maetzler, MA[a], Anja Mehnert-Theuerkauf, PhD[c], Felipe A. Montellano, MD[n,o,u], Caroline Morbach, MD[u,v], Sein Schmidt, MD[w], Jan Heyckendorf, MD[e,f], Flo Steigerwald[b], Stefan Stoerk, MD, PhD[u,v], Christina Lemhoefer MD[x], Stefan Schreiber, MD[e], Carsten Finke, MD[b†] Walter Maetzler, MD[a†]

a *Neurology Department, University Medical Center Schleswig-Holstein, Campus Kiel, Arnold-Heller-Str. 3, 24105 Kiel, Germany*

b *Charité-Universitätsmedizin Berlin, Department of Neuroloy and Experimental Neurology, Bonhoefferweg 3, 10117 Berlin, Germany*

c *Department of Medical Psychology and Medical Sociology, University Medical Center Leipzig, Philipp-Rosenthal-Str. 55, 04103 Leipzig Germany*

d *Department of general Psychologie I and Biological Psychology, Christian-Albrechts-Universität zu Kiel, Neufeldstraße 4a, 24118 Kiel, Germany*

e *Internal Medicine Department I, Leibniz Lung Clinic, University Hospital Schleswig Holstein, Campus Kiel, Arnold-Heller-Str. 3, 24105 Kiel, Germany*

f *Airway Research Center North (ARCN), German Center for Lung Research (DZL), Woehrendamm 80, 22927 Grosshansdorf, Germany*

g *Institute for Epidemiology, Christian-Albrechts-University Kiel, Niemanndweg 11, 24105 Kiel, Germany*

h *Department of Medical Informatics, University Medical Center Goettingen, Von-Siebold-Str. 3, 37075 Goettingen, Germany*

i *Center for Stroke Research Berlin, Charitéplatz 1, 10117 Berlin, Germany*

j *Excellence Cluster NeuroCure, Charitéplatz 1, 10117 Berlin, Germany*

k *German Center for Neurodegenerative Diseases (DZNE), partner site Berlin, Charitéplatz 1, 10117 Berlin, Germany*

l *German Centre for Cardiovascular Research (DZHK), partner site Berlin, Charitéplatz 1, 10117 Berlin, Germany*

m *German Centre for mental health (DZPG), partner site Berlin, Charitéplatz 1, 10117 Berlin, Germany*

n *University of Ulm, Department of Neurology, Oberer Eselsberg 45, 89081 Ulm, Germany*

o *University of Wuerzburg, Institute of Clinical Epidemiology and Biometry, Josef-Schneider-Str. 2, 97080 Wuerzburg, Germany*

p *University Hospital Wuerzburg, Clinical Trial Center, Oberduerrbacher Str. 6, 97080 Wuerzburg, Germany*

q *University Hospital Wuerzburg, Institute for medical Data Science, Josef-Schneider-Str. 2, 97080 Wuerzburg, Germany*

r *University of Cologne, Faculty of Medicine and University Hospital Cologne, Department I of Internal Medicine, Center for Integrated Oncology Aachen Bonn Cologne Duesseldorf, Kerpener Str. 62, Germany*

s *Institute of Medical Informatics and Statistics, Kiel University, University Medical Center Schleswig-Holstein Campus Kiel, Arnold-Heller-Str. 3, 50937 Koeln, Germany*

t *Institute of Social Medicine, Epidemiology and Health Economics, Charité-Universitätsmedizin Berlin, Schumannstr. 20, 10117 Berlin, Germany*

u *University Hospital Wuerzburg, Department Clinical Research and Epidemiology, Comprehensive Heart Failure Center, Am Schwarzenberg 15, 97080 Wuerzburg, Germany*

v *University Hospital Wuerzburg, Department for Medicine I, Oberduerrbacher Str. 6, 97080 Wuerzburg, Germany*

w *Berlin Institute of Health at Charité – Universitätsmedizin Berlin, Clinical Study Center, Anna-Louisa-Karsch-Straße 2, 10178 Berlin, Germany*

x *Institute of Physical and Rehabilitation Medicine, Jena University Hospital/Friedrich-Schiller-University Jena, Am Klinikum 1, 07747 Jena, Germany*

[*] contributed equally as first authors

[†] contributed equally as senior authors

Corresponding author

M. Sc. Christian Neumann
Klinik fuer Neurologie
Universitätsklinikum Schleswig-Holstein, Campus Kiel
Arnold-Heller-Straße 3
24105 Kiel
Germany

Phone: ++49+30-450-560216
E-mail: Christian.Neumann@uksh.de

ORCID iD: 0000-0002-5891-1937

Supplementary Table S1

Independent variables used in the recursive feature elimination and how they were measured.

Variables	Measures
**Fatigue**	**FACIT-Fatigue Scale**
**Personal stress**	**PSS total score**
Cognitive functioning	MoCA total score
Subjective cognitive decline	Yes, No
Subjective word finding disorder	Yes, No
**Sex**	**Male, Female**
**Age**	**Years**
BMI	Weight, Height
**Employment (At follow-up: Loss of employment)**	**Employed, Not employed (Yes, No)**
Partner	Yes, No
Vaccinated	Yes, No
More than 12 years education	Yes, No
Treatment during initial SARS-CoV-2 infection	Yes, No
Treatment at home	Yes, No
Stationary treatment	Yes, No
Treatment in intensive care unit	Yes, No
Treatment with ventilation	Yes, No
Initial infection symptoms	Number of symptoms during initial SARS-CoV-2 infection
Time since infection	Days between initial SARS-CoV-2 infection and assessment
Pre-diagnosed diseases	
Any neurological/psychiatric disease	Yes, No
COPD	Yes, No
Sleep apnea	Yes, No
Tumor disease	Yes, No
Depression	Yes, No
Anxiety	Yes, No
Migraine	Yes, No
**Remaining symptoms**	**Number of symptoms remaining from initial SARS-CoV-2 infection**
**Smelling disturbance**	**Yes, No**
**Impaired sense of taste**	**Yes, No**
**Stomach pain**	**Yes, No**
**Confusion**	**Yes, No**
**Diarrhea**	**Yes, No**
**Vomiting**	**Yes, No**
**Nausea**	**Yes, No**
**Vertigo**	**Yes, No**
**Coughing**	**Yes, No**
**Hoarseness**	**Yes, No**
**Sore throat**	**Yes, No**
**Running nose**	**Yes, No**
**Chills**	**Yes, No**
**Muscle pain**	**Yes, No**
**Joint pain**	**Yes, No**
**Shortness of breath**	**Yes, No**
**Wheezing**	**Yes, No**
**Chest pain**	**Yes, No**
**Rash**	**Yes, No**
**Fever**	**Yes, No**
**Headache**	**Yes, No**
**Hair loss**	**Yes, No**

Note. Highlighted variables were also selected for the follow-up Analyses. BMI, Body-Mass-Index; EQ-5D-5L, European Quality of Life 5 Dimensions 5 Level Version; FACIT-Fatigue Scale, Functional Assessment of Chronic Fatigue Illness – Fatigue Subscore; MoCA, Montreal Cognitive Assessment; PSS, Perceived Stress Scale.

Supplementary Table S2

Sociodemographic and clinical characteristics of the COVIDOM study sample at baseline.

Characteristics [N]	Baseline = 3475		Follow-Up = 2510	
**sex**	**Missing = 2**			
Female [N (%)]	1944 (56)		1422 (57)	
Male [N (%)]	1529 (44)		1088 (43)	
**Age [years]**				
18-34 [N (%)]	1078 (31)		712 (28)	
35-49 [N (%)]	952 (27)		652 (26)	
50-64 [N (%)]	1091 (31)		851 (34)	
65-88 [N (%)]	354 (10)		295 (12)	
**Unemployment**				
Yes [N (%)]	628 (18)		-	
**BMI**	**Missing = 39**			
Underweight [N (%)]	44 (1)		-	
Normal weight [N (%)]	1392 (40)		-	
Overweight [N (%)]	1164 (33)		-	
Obese [N (%)]	836 (24)		-	
**Time between initial SARS-CoV-2 infection and baseline**				
6 – 9 months [N (%)]	1618 (47)		-	
9 – 12 months [N (%)]	1334 (38)		-	
≥12 months [N (%)]	523 (15)		-	
**Number of initial infection symptoms**			**Missing = 30**	
Asymptomatic [N (%)]	306 (9)		202 (8)	
1 – 5 [N (%)]	528 (15)		396 (16)	
6 – 8 [N (%)]	701 (20)		493 (20)	
9 – 11 [N (%)]	783 (23)		560 (22)	
12 – 21 [N (%)]	1157 (33)		829 (33)	
**Number of remaining symptoms**				
Asymptomatic [N (%)]	1912 (55)		1433 (57)	
1 – 5 [N (%)]	1353 (39)		974 (39)	
6 – 8 [N (%)]	155 (4)		74 (3)	
9 – 11 [N (%)]	43 (1)		24 (1)	
12 – 21 [N (%)]	12 (< 1)		5 (< 1)	
**Clinically relevant fatigue (FACIT-Fatigue Scale)**	**Missing = 266**		**Missing = 91**	
Yes	689 (20)		444 (18)	
**Pre-diagnosed COVID-19 comorbidity**		Missings		Missings
Any neurological/ psychiatric disorder	841 (24)	11	602	110
Depression	387 (11)	113	290	101
Migraine	309 (10)	92	220	103
Anxiety	108 (3)	94	71	83
Apnea	146 (4)	70	113	101

COPD	53 (1)	92	38	73
Tumor	48 (1)	58	30	40
**EQ-5D-5L "mobility"**	**Missing = 176**		**Missing = 62**	
No problems	2544 (73)		1961 (78)	
Slight problems	459 (13)		293 (12)	
Moderate problems	220 (6)		150 (6)	
Major problems	73 (2)		44 (2)	
Extreme problems	3 (< 1)		0 (0)	
**EQ-5D-5L "self-care"**	**Missing = 172**		**Missing = 62**	
No problems	3168 (91)		2348 (94)	
Slight problems	91 (3)		67 (3)	
Moderate problems	29 (< 1)		23 (1)	
Major problems	10 (< 1)		9 (< 1)	
Extreme problems	5 (< 1)		1 (< 1)	
**EQ-5D-5L "usual activities"**	**Missing = 177**		**Missing = 66**	
No problems	2036 (59)		1647 (66)	
Slight problems	756 (22)		522 (21)	
Moderate problems	338 (10)		176 (7)	
Major problems	150 (4)		89 (4)	
Extreme problems	18 (< 1)		10 (< 1)	
**EQ-5D-5L "pain/discomfort"**	**Missing = 170**		**Missing = 63**	
No problems	1478 (43)		1306 (52)	
Slight problems	1073 (31)		684 (27)	
Moderate problems	553 (16)		339 (14)	
Major problems	186 (5)		108 (4)	
Extreme problems	15 (< 1)		10 (< 1)	
**EQ-5D-5L "anxiety/depression"**	**Missing = 178**		**Missing = 63**	
No problems	2041 (59)		1614 (64)	
Slight problems	802 (23)		556 (22)	
Moderate problems	294 (8)		179 (7)	
Major problems	132 (4)		80 (3)	
Extreme problems	28 (< 1)		18 (< 1)	

Note. BMI, Body-Mass-Index; COPD, chronic obstructive pulmonary disease; EQ-5D-5L, European Quality of Life 5 Dimensions 5 Level Version; FACIT-Fatigue Scale, Functional Assessment of Chronic Fatigue Illness – Fatigue Subscore

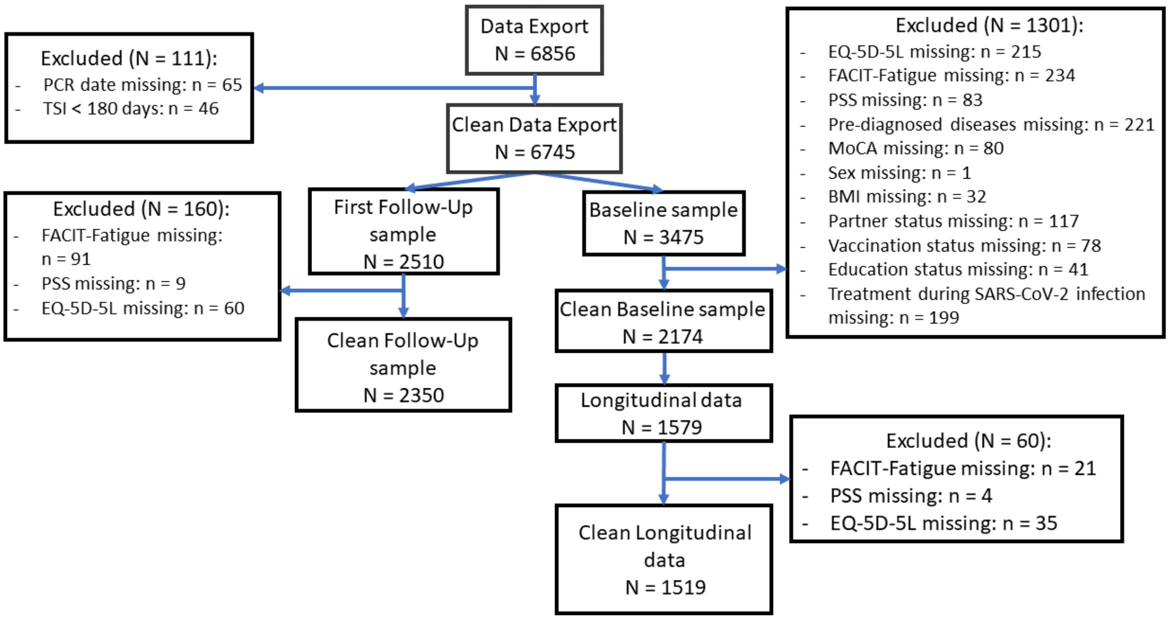

Supplementary Fig. S3 Steps and sequence of data cleaning for the COVIDOM study samples. BMI, Body-Mass-Index; EQ-5D-5L, European Quality of Life 5 Dimensions 5 Level Version; FACIT-Fatigue Scale, Functional Assessment of Chronic Fatigue Illness – Fatigue Subscore; MoCA, Montreal Cognitive Assessment; PSS, Perceived Stress Scale; TSI, Time since infection

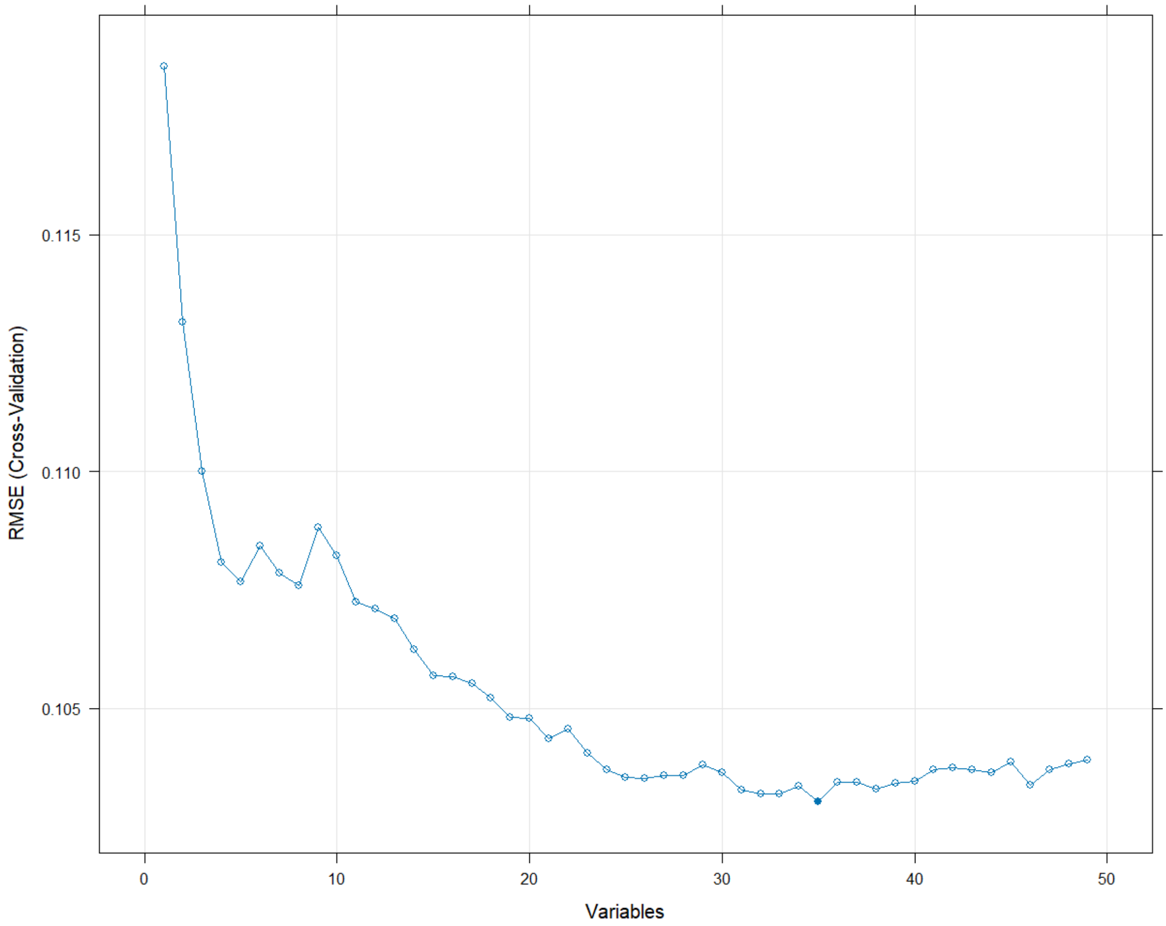

Supplementary Fig. S4 Root mean square errors (RMSE) of the models calculated by recursive feature elimination using the European Quality of Life 5 Dimensions 5 Level Version (EQ-5D-5L) index at baseline as the dependent variable. It shows the model fit in dependence of the number of selected features. The dark-blue point is the number of features with the lowest RMSE. A 35-variable model was calculated as the model with the lowest RMSE of 0.1 to explain the EQ-5D-5L index. We chose a 5-variable model as the final model because the RMSE with 5 variables is only 0.005 less than the RMSE with 35 variables, while using significantly fewer variables

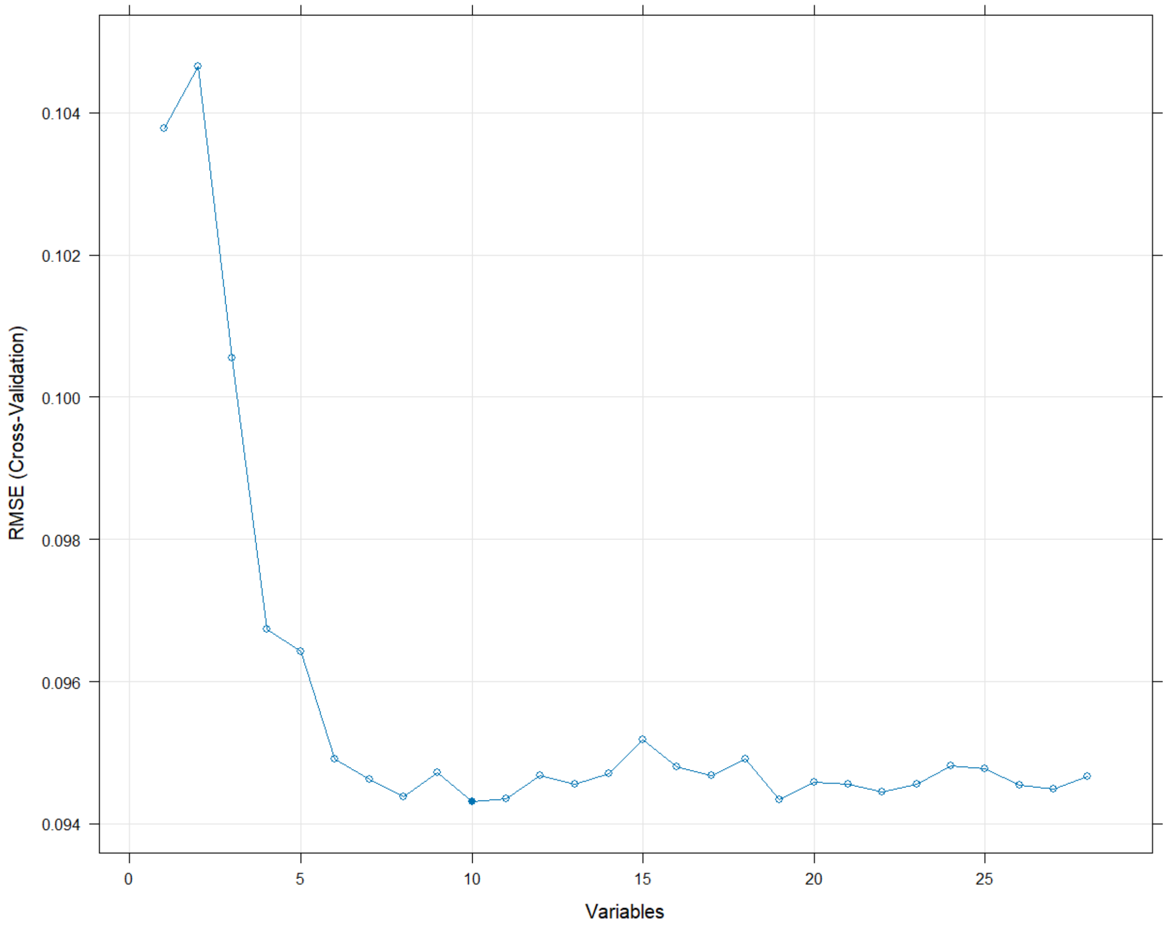

Supplementary Fig. S5 Root mean square errors (RMSE) of the models calculated by recursive feature elimination using the European Quality of Life 5 Dimensions 5 Level Version (EQ-5D-5L) index at follow-up as the dependent variable. It shows the model fit in dependence of the number of selected features. The dark-blue point is the number of features with the lowest RMSE. A 10-variable model as the model with the lowest RMSE of 0.09 to explain the EQ-5D-5L index. We chose a 6-variable model as the final model because the RMSE with 6 variables was also 0.09, while using significantly fewer variables

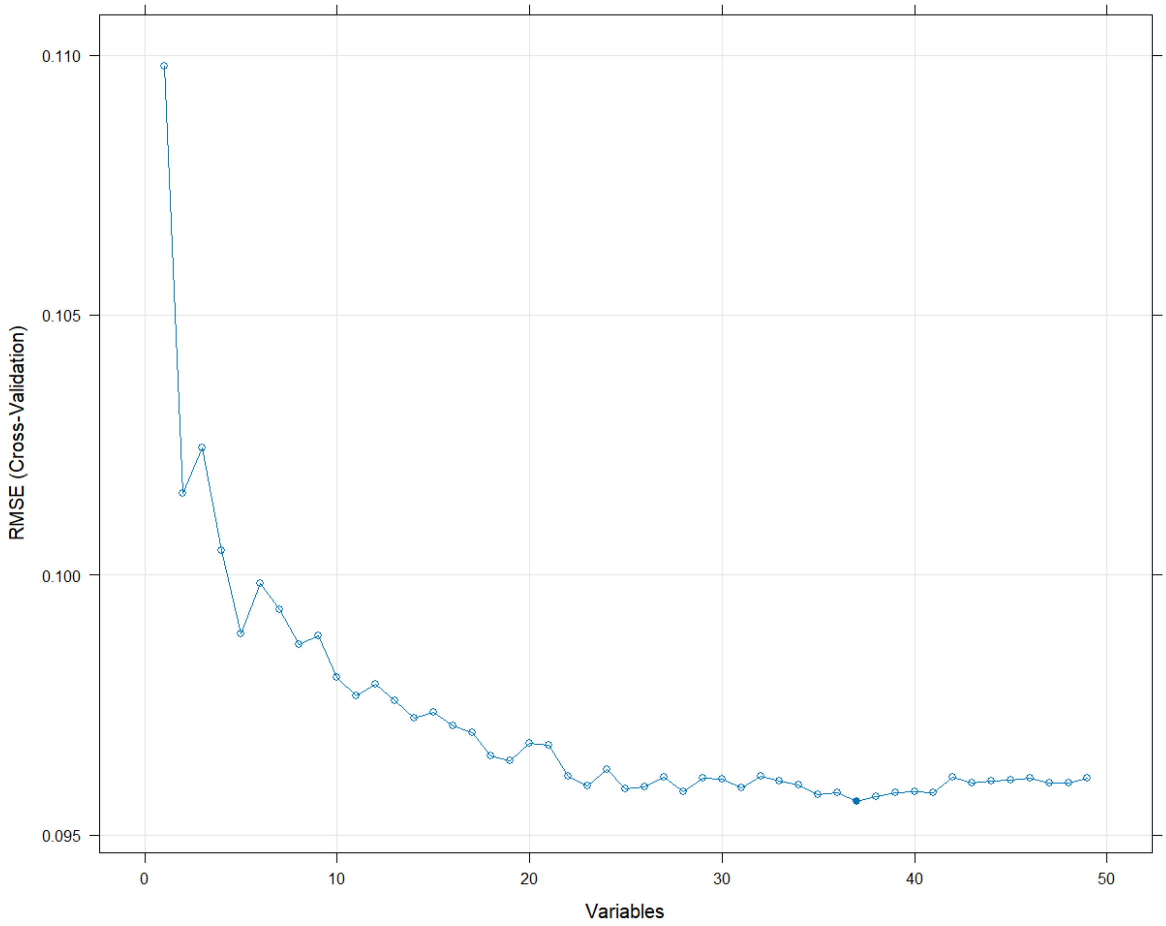

Supplementary Fig. S6 Root mean square errors (RMSE) of the models calculated by recursive feature elimination using the European Quality of Life 5 Dimensions 5 Level Version (EQ-5D-5L) index at baseline as the dependent variable for participants without a pre-diagnosed anxiety or depression. It shows the model fit in dependence of the number of selected features. The dark-blue point is the number of features with the lowest RMSE. A 37-variable model as the model with the lowest RMSE of 0.1 to explain the EQ-5D-5L index. We chose a 5-variable model as the final model because the RMSE with 6 variables was still 0.1, while using significantly fewer variables

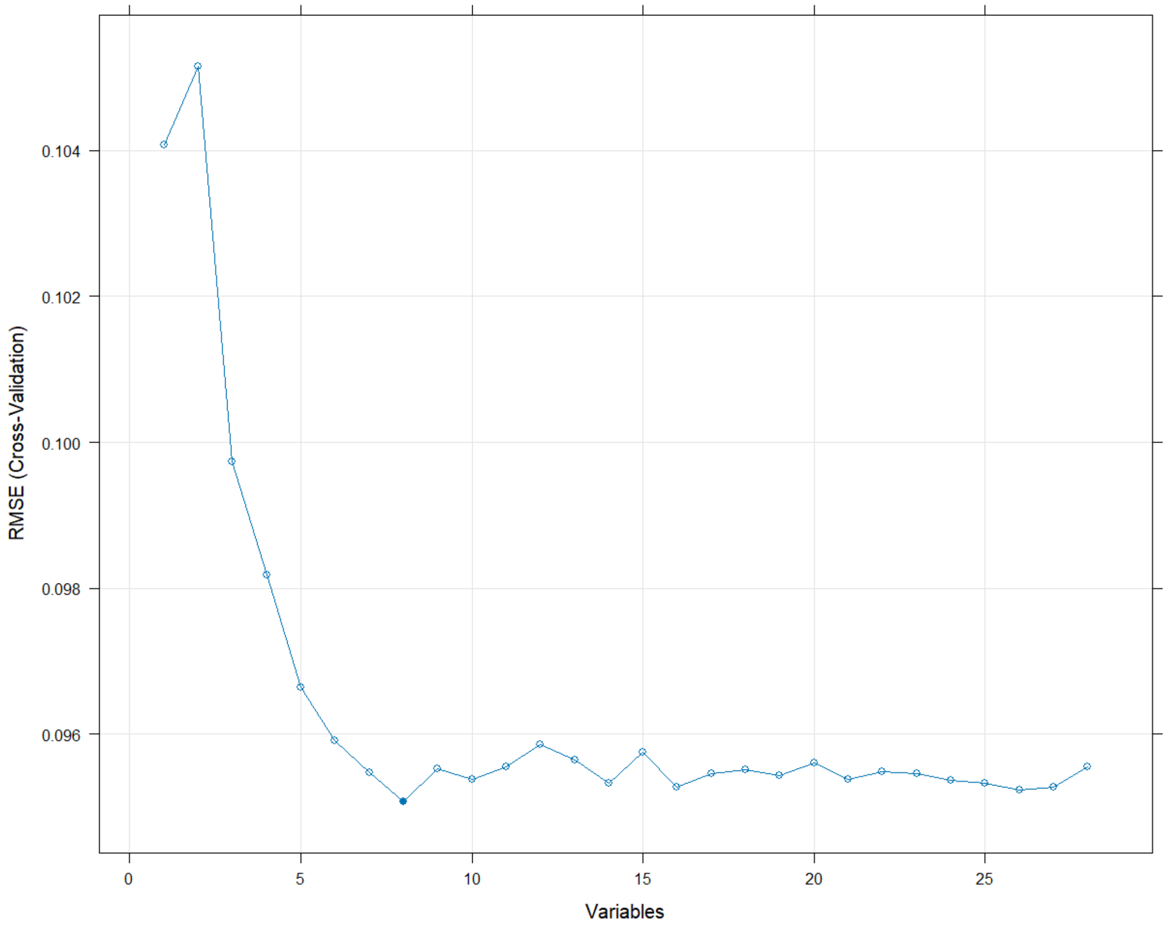

Supplementary Fig. S7 Root mean square errors (RMSE) of the models calculated by recursive feature elimination using the European Quality of Life 5 Dimensions 5 Level Version (EQ-5D-5L) index at follow-up as the dependent variable for participants without a pre-diagnosed anxiety or depression. It shows the model fit in dependence of the number of selected features. The dark-blue point is the number of features with the lowest RMSE. An 8-variable model as the model with the lowest RMSE of 0.10 to explain the EQ-5D-5L index. We chose a 6-variable model as the final model because the RMSE with 6 variables was only 0.001 less than the RMSE with 8 variables, while using significantly fewer variables

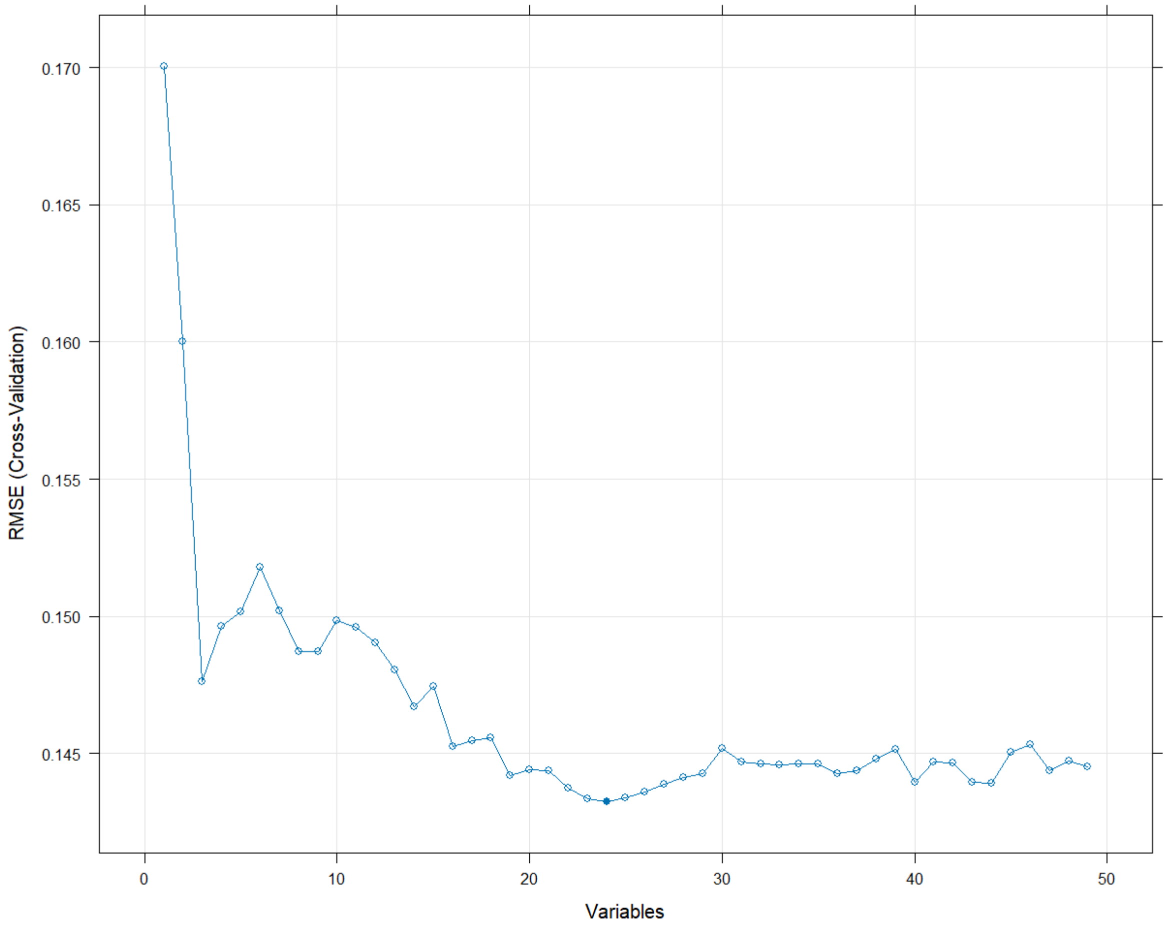

Supplementary Fig. S8 Root mean square errors (RMSE) of the models calculated by recursive feature elimination using the European Quality of Life 5 Dimensions 5 Level Version (EQ-5D-5L) index at baseline as the dependent variable for participants with a pre-diagnosed anxiety or depression. It shows the model fit in dependence of the number of selected features. The dark-blue point is the number of features with the lowest RMSE. A 24-variable model as the model with the lowest RMSE of 0.14 to explain the EQ-5D-5L index. We chose a 3-variable model as the final model because the RMSE with 3 variables was only 0.004 lower than the RMSE with 24 variables, while using significantly fewer variables

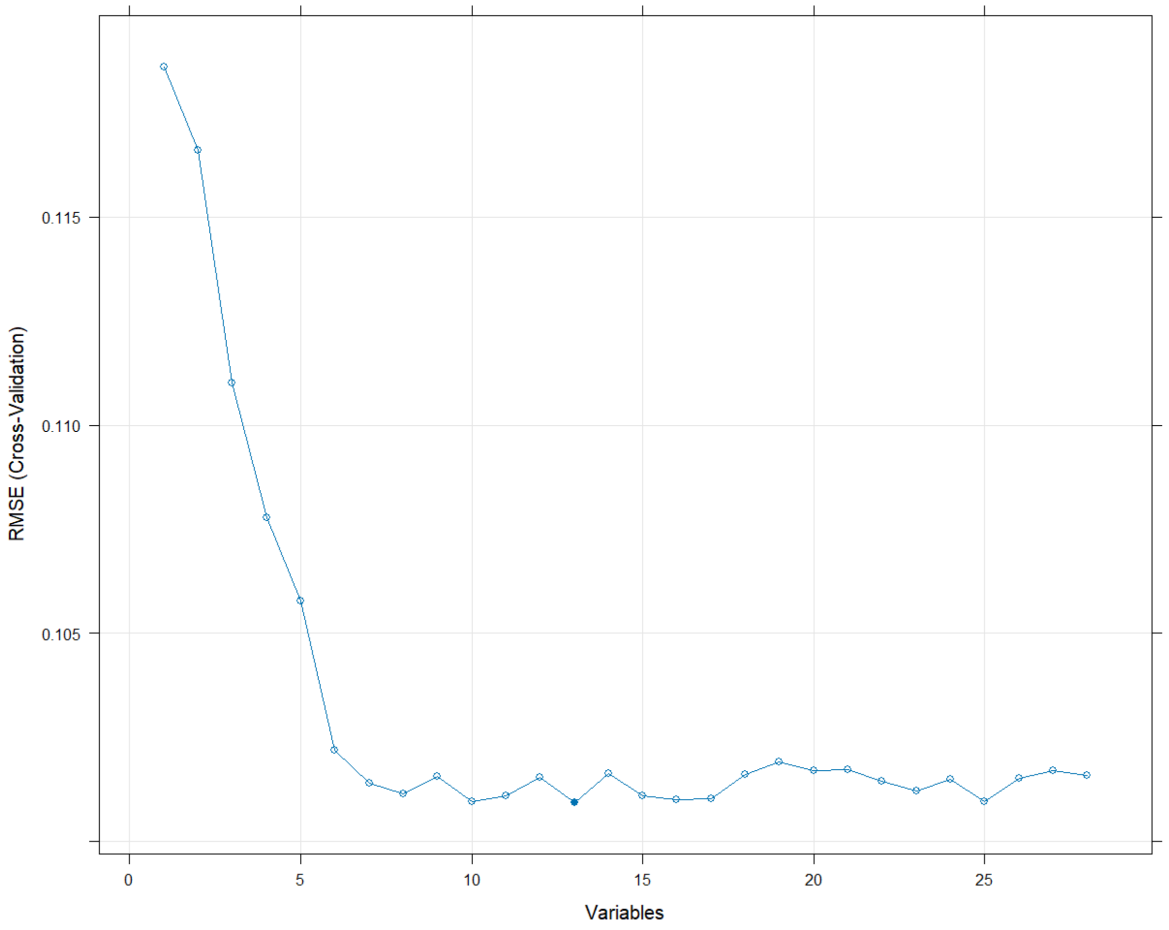

Supplementary Fig. S9 Root mean square errors (RMSE) of the models calculated by recursive feature elimination using the European Quality of Life 5 Dimensions 5 Level Version (EQ-5D-5L) index at follow-up as the dependent variable for participants with pre-diagnosed anxiety or depression. It shows the model fit in dependence of the number of selected features. The dark-blue point is the number of features with the lowest RMSE. A 13-variable model as the model with the lowest RMSE of 0.10 to explain the EQ-5D-5L index. We chose a 6-variable model as the final model because the RMSE with 6 variables was still 0.10, while using significantly fewer variables

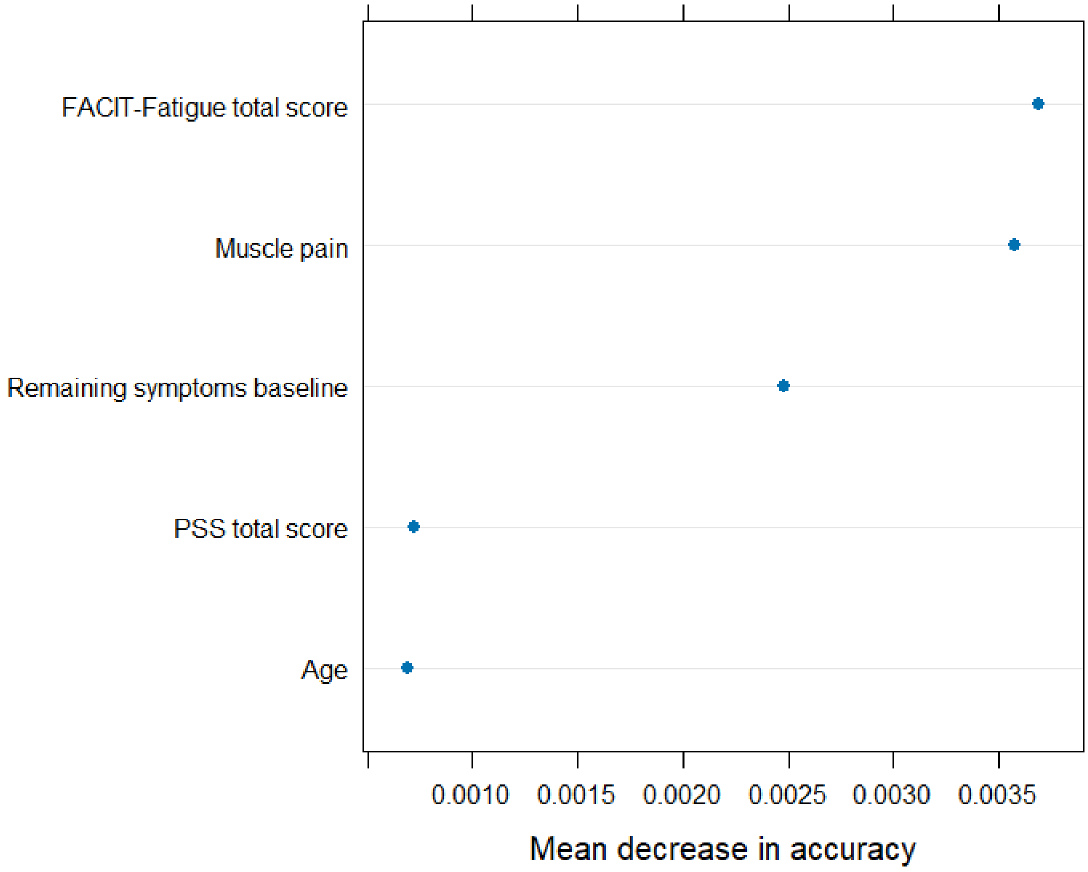

Supplementary Fig. S10 Mean decrease in accuracy of the selected variables at baseline after a random forest regression on the training set with the European Quality of Life 5 Dimensions 5 Level Version index as the dependent variable. FACIT-Fatigue Scale, Functional Assessment of Chronic Fatigue Illness – Fatigue Subscore; PSS, Perceived Stress Scale

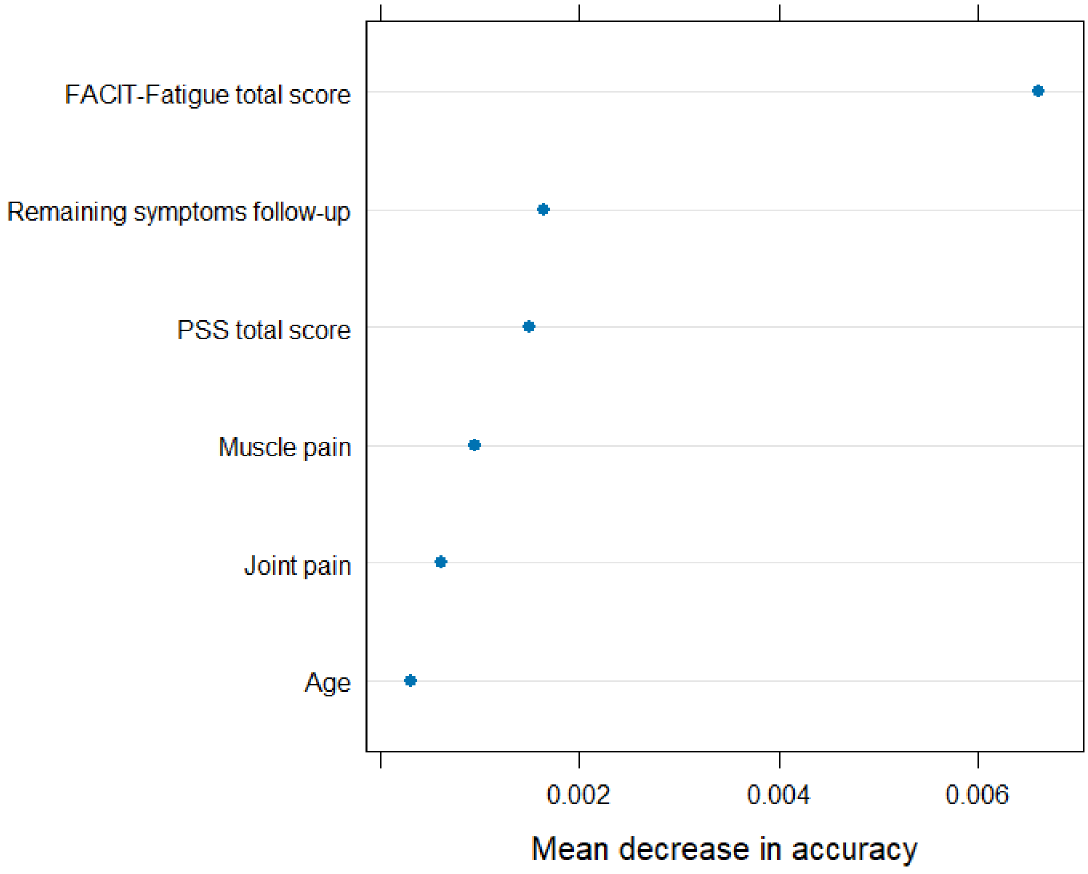

Supplementary Fig. S11 Mean decrease in accuracy of the selected variables at follow-up after a random forest regression on the training set with the European Quality of Life 5 Dimensions 5 Level Version index as the dependent variable. FACIT-Fatigue Scale, Functional Assessment of Chronic Fatigue Illness – Fatigue Subscore; PSS, Perceived Stress Scale

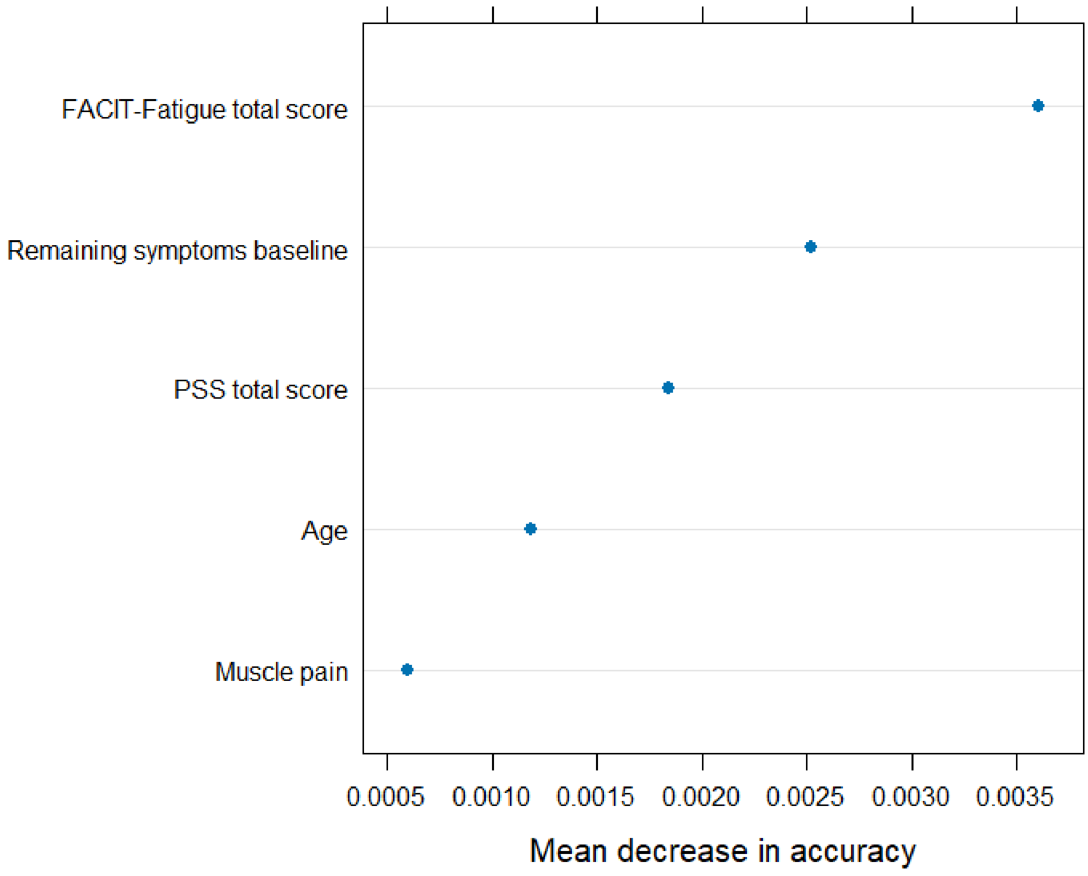

Supplementary Fig. S12 Mean decrease in accuracy of the selected variables at baseline after a random forest regression on the training set with the European Quality of Life 5 Dimensions 5 Level Version index as the dependent variable for participants without a pre-diagnosed anxiety or depression. FACIT-Fatigue Scale, Functional Assessment of Chronic Fatigue Illness – Fatigue Subscore; PSS, Perceived Stress Scale

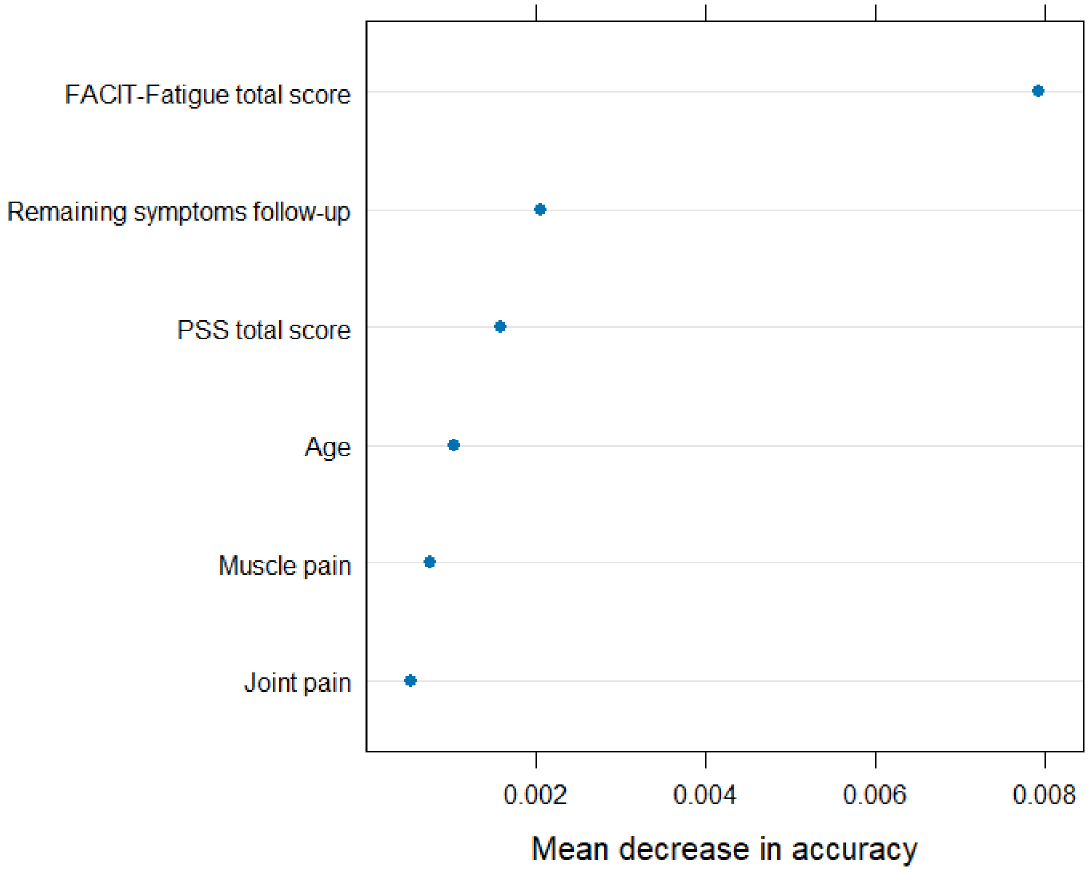

Supplementary Fig. S13 Mean decrease in accuracy of the selected variables at follow-up after a random forest regression on the training set with the European Quality of Life 5 Dimensions 5 Level Version index as the dependent variable for participants without a pre-diagnosed anxiety or depression. FACIT-Fatigue Scale, Functional Assessment of Chronic Fatigue Illness – Fatigue Subscore; PSS, Perceived Stress Scale

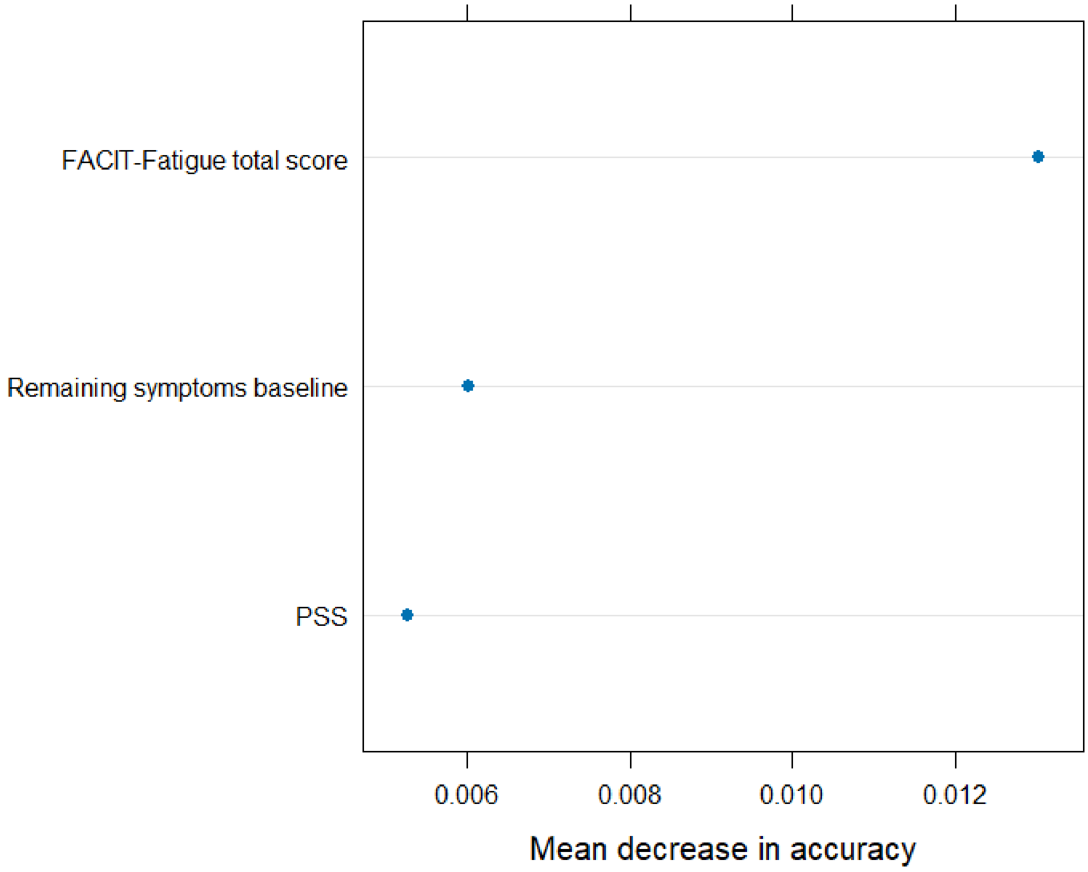

Supplementary Fig. S14 Mean decrease in accuracy of the selected variables at baseline after a random forest regression on the training set with the European Quality of Life 5 Dimensions 5 Level Version index as the dependent variable for participants with a pre-diagnosed anxiety or depression. FACIT-Fatigue Scale, Functional Assessment of Chronic Fatigue Illness – Fatigue Subscore; PSS, Perceived Stress Scale

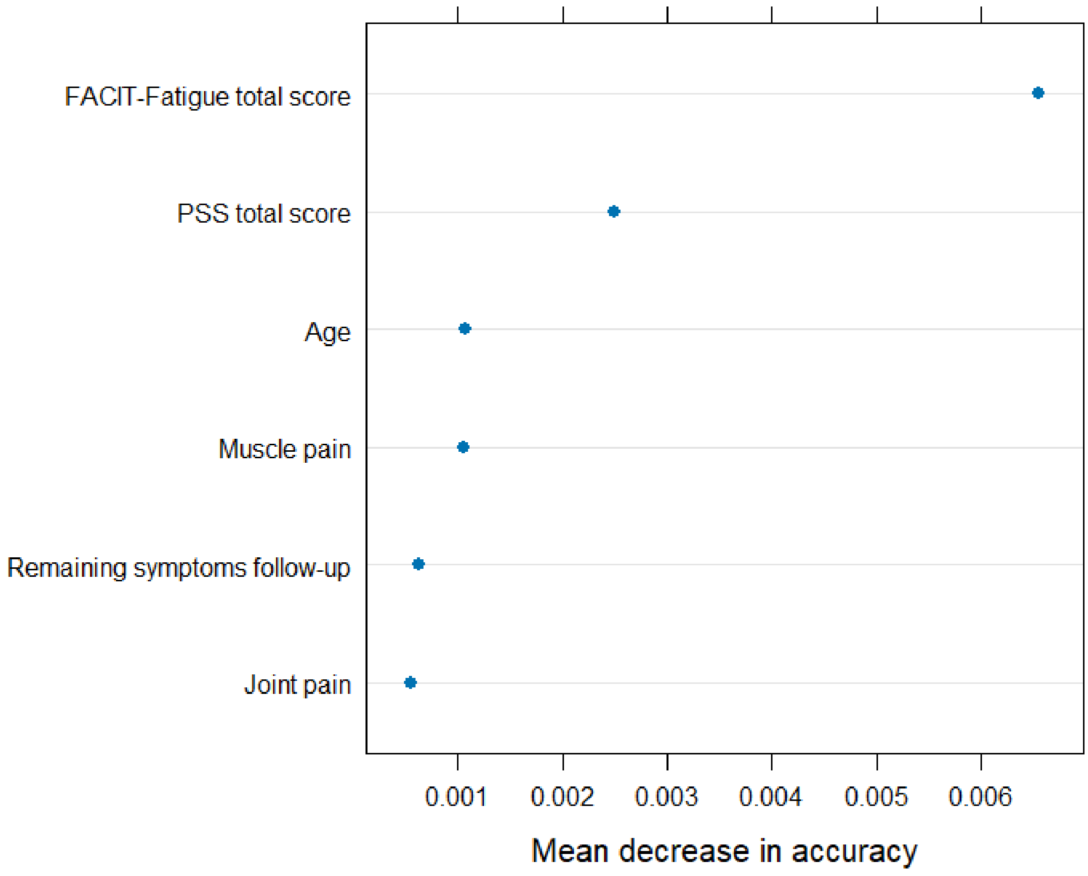

Supplementary Fig. S15 Mean decrease in accuracy of the selected variables at follow-up after a random forest regression on the training set with the European Quality of Life 5 Dimensions 5 Level Version index as the dependent variable for participants with pre-diagnosed anxiety or depression. FACIT-Fatigue Scale, Functional Assessment of Chronic Fatigue Illness – Fatigue Subscore; PSS, Perceived Stress Scale

Supplementary Table S16

Order of selected variables after recursive feature elimination for each final random forest regression model at baseline and follow-up.

EQ-5D-5L	During Baseline			During Follow-Up		
	Relevant independent variables	R^2 [%]	RMSE	Relevant independent variables	R^2 [%]	RMSE
Index	1. FACIT-Fatigue Scale<br>2. Muscle pain<br>3. Remaining symptoms<br>4. PSS<br>5. Age	39.22	0.11	1. FACIT-Fatigue Scale<br>2. Remaining symptoms<br>3. PSS<br>4. Muscle pain<br>5. Joint pain<br>6. Age	54.07	0.10
Index – participants without a pre-diagnosed neurological/ psychiatric disorder	1. FACIT-Fatigue Scale<br>2. Remaining symptoms baseline<br>3. PSS<br>4. Age<br>5. Muscle pain	34.54	0.09	1. FACIT-Fatigue Scale<br>2. Remaining symptoms follow-up<br>3. PSS<br>4. Age<br>5. Muscle pain<br>6. Joint pain	43.26	0.09
Index – participants with at least one pre-diagnosed neurological/ psychiatric disorder	1. FACIT-Fatigue Scale<br>2. Remaining symptoms baseline<br>3. PSS	30.22	0.15	1. FACIT-Fatigue Scale<br>2. PSS<br>3. Age<br>4. Muscle pain<br>5. Remaining symptoms<br>6. Joint pain	53.25	0.11

Note. EQ-5D-5L, European Quality of Life 5 Dimensions 5 Level Version; FACIT-Fatigue Scale, Functional Assessment of Chronic Fatigue Illness – Fatigue Subscore; PSS, Perceived Stress Scale

Supplementary Table S17

Coefficient of determination (R^2) and root mean square error (RMSE) for each dimension of the EQ-5D-5L at baseline and follow-up

EQ-5D-5L	During Baseline		During Follow-Up	
	R^2 [%]	RMSE	R^2 [%]	RMSE
Mobility	28.58	0.61	23.43	0.59
Self-Care	3.68	0.31	13.60	0.29
Usual activities	47.12	0.62	51.04	0.55
Pain/discomfort	37.76	0.72	39.56	0.70
Anxiety/depression	29.37	0.71	43.00	0.62

Note. Calculated using random forest regression models with FACIT-Fatigue Scale, age, PSS, number of symptoms remaining from initial SARS-CoV-2 infection, and muscle pain as independent variables. EQ-5D-5L, European Quality of Life 5 Dimensions 5 Level Version; FACIT-Fatigue Scale, Functional Assessment of Chronic Fatigue Illness – Fatigue Subscore; PSS, Perceived Stress Scale

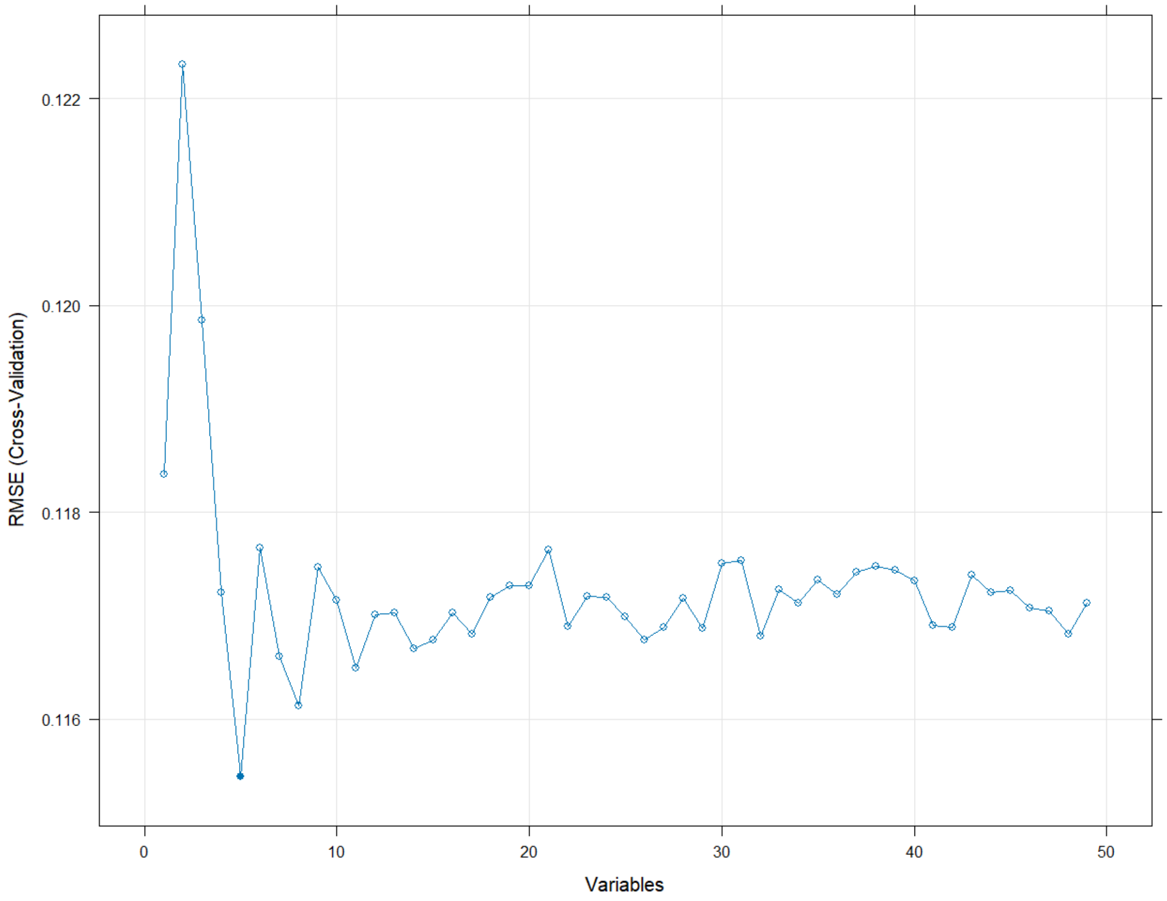

Supplementary Fig. S18 Root mean square errors (RMSE) of the models calculated by recursive feature elimination predicting the European Quality of Life 5 Dimensions 5 Level Version EQ-5D-5L index at follow-up using baseline variables. It shows the model fit in dependence of the number of selected features. The dark-blue point is the number of features with the lowest RMSE. A 5-variable model was calculated as the model with the lowest RMSE of 0.12 to explain the EQ-5D-5L index.
